# From Surviving to Thriving: A Trauma-Informed Yoga Intervention for Adolescents and Educators in Rural Montana

**DOI:** 10.3390/educsci14121394

**Published:** 2024-12-19

**Authors:** Lauren Davis, Brandon G. Scott, Greta M. Linse, Rebecca Buchanan

**Affiliations:** 1 Department of Education, Montana State University, Bozeman, MT 59717, USA; 2 Department of Psychology, Montana State University, Bozeman, MT 59717, USA; 3 Department of Mathematics, Montana State University, Bozeman, MT 59717, USA; 4 Department of Health and Human Performance, Emory & Henry College, Emory, VA 24327, USA

**Keywords:** trauma, mental health, rural schools, intervention, social emotional learning

## Abstract

**Background:**

Due to the mental health crisis that has spiraled since the onset of COVID-19, particularly among the nation’s youth, the purpose of this study was to examine the efficacy of a novel, school-based mental health intervention for high school students (ages 15–17 years). This project’s main aim was to determine which intervention modality was more effective with students across two school districts with varying degrees of rurality (in-person delivery vs. remote delivery). A secondary aim of this study was to determine the efficacy of a remotely delivered, concurrent intervention for educators across both school districts. This study took place in rural southwestern Montana.

**Methods:**

Utilizing a 6-week, trauma-informed yoga intervention, comparisons of mental and physical health outcomes were performed using cohort data drawn from participants’ physiological data and validated mental health survey measures.

**Results:**

While physiological results were mixed across experimental groups, mental health outcomes were overwhelmingly positive for all groups. Additionally, educators reported improvements in career satisfaction and burnout levels.

**Conclusions:**

Findings indicate a great deal of promise with this intervention in improving mental health outcomes for both students and educators. Moreover, a face-to-face intervention for students showed dramatic improvement in physiological stress indicators.

## 1. Introduction

Since the onset of the COVID-19 pandemic, the mental health of youth has been and continues to be an area of concern, particularly in educational contexts. Data indicate that student mental health is a rising epidemic as the number of high school students who feel sad or hopeless has continued to increase [1]. In 2009, according to the Youth Risk Behavior Survey, the percentage of students feeling sad or hopeless almost every day for at least two weeks was 26.9%. In 2021, the percentage increased to 42.3% [1]. When analyzing the data based on gender, the percentage reported was much higher for females (56.6%) than males (28.6%). A meta-analysis also mirrored the same findings, indicating an increase in depression and anxiety symptoms [2]. In terms of high school students who seriously considered or attempted suicide, the numbers also increased from 13.8% (seriously considered) and 6.3% (attempted) in 2009 to 22.2% (seriously considered) and 10.2% (attempted) in 2021 [1]. These data underscore the increasing severity of the mental health crisis among our nation’s youth, which also has implications for physical health as well [3,4]. Of particular relevance to this study is a noted gap in the literature related to mental health interventions in rural communities and school contexts. Thus, this project seeks to address this gap. The study’s primary aim was to determine which intervention modality was more effective with students across two rural school districts with varying degrees of rurality (in-person delivery vs. remote delivery). A secondary aim of this study was to determine the efficacy of a remotely delivered, concurrent intervention for educators across both school districts.

### 1.1. The Effects of Trauma on Adolescent Well-Being

Even prior to the pandemic, mental health challenges were one of the leading causes of negative life outcomes for young people [5]. Along with the effects of this widespread contagion, the pandemic also revealed that the mental health crisis was disproportionately impacting minority populations including marginalized individuals who have experienced poverty and trauma [5]. The related stress and adverse childhood experiences of marginalized youth, such as those living in rural areas, can negatively impact brain development [6]. The stress response or activation of the autonomic nervous system due to traumatic experiences can create challenges related to memory and information processing. According to Gross (2020), “real, measurable, negative physiological changes, including those in the brain, take place when the autonomic nervous system is repeatedly put into a state of high alert or, worse, is regularly in that state” [6] (p. 50). During childhood, experiences shape neural plasticity in the brain. Both positive and negative experiences throughout one’s youth can shape the trajectory of a child’s mental and physical health [7]. Due to the developing nature of a child’s brain, toxic stress not only impacts the internal structures of the brain, including the hippocampus (the brain’s memory center) and amygdala (the brain’s fight/flight/freeze area), but it also affects other body systems [8,9]. Such examples of additional physiological impacts of childhood adversity include impacts on one’s cortisol (stress hormone) levels and vagal nerve functioning, as well as decreased heart rate variability [10,11]. Germain to this study, a child’s environment and context can also play a crucial role in exposure to adversity and accessibility to supportive healthcare services for mental and physical well-being.

### 1.2. Study Context: Rural Montana

This study took place in rural southwestern Montana across two different school districts: one located in a town with roughly 8500 residents and the other in a remote location with fewer than 800 year-round residents. In the more rural of the two locations, residents face a minimum 60-mile drive to the nearest hospital (located in the first school district) and roughly 175 miles to the state’s only children’s hospital that offers acute mental health support for youth. These two locations provide a lens into the difficulties rural Montanans face in terms of access to quality healthcare providers. Not only are these providers at a great distance from the more remote study site, but dangerous weather also often creates an additional barrier; frequent winter snowstorms close roads regularly, and in 2022, this community became completely cut off due to roads being washed away in a catastrophic flood. Therefore, participants based in this location not only face geographic challenges to quality mental healthcare, but they have also potentially experienced the trauma related to a natural disaster (which led to the loss of multiple homes and employment in the area) [12]. Further, the rurality of the study context necessitated examining a remote delivery option; there are no trauma-informed yoga instructors located in the more rural of the two school districts, so offering a remotely delivered intervention was the only feasible option for the school’s participation. A remote delivery option was also intended to be of particular benefit to the educator participants so that they could exercise flexibility in terms of where they participated in order to accommodate their varying schedules. Finally, testing a remotely delivered intervention also has larger implications for scalability and intervention access for additional remote study sites in future iterations of this research.

Additionally, the cultural context of rurality plays an important role in mental health. Montanans often embrace the “cowboy up” culture, which emphasizes rugged individualization and self-reliance; however, this mentality often stigmatizes seeking mental health support as a “weakness” [13–15]. Given that childhood adversity is more prevalent in rural America than in its urban counterparts, and especially given the suicide crisis in Montana specifically, it is particularly critical to de-stigmatize mental healthcare to best support its vulnerable youth [16–20].

### 1.3. The Importance of Educator Wellness

While this study focused on supporting the mental and physical well-being of rural youth in Montana, a secondary lynchpin of this project was to concurrently support the educators responsible for these adolescents daily. Patricia Wilcox, a licensed clinical social worker, is quoted as asserting “if we [school/district administrators] do not feed the staff, they’ll eat the children” (2023, personal communication). In other words, if the adults who provide care for adolescents in school classrooms each day are not in a good frame of mind, their attitudes will negatively impact their students. Established research suggests that when educators are emotionally dysregulated, their students are more stressed and exhibit lower academic achievement [21]. Conversely, improvements in educator well-being and educator reductions in depressive symptomatology are associated with improvements in student psychological well-being [21]. Therefore, our study utilized a holistic approach to support student mental health by including our student participants’ educators in a concurrent mental health intervention.

### 1.4. Research Orientation and Intervention Framework: Trauma-Informed Yoga

As mentioned above, context matters; in this study, we utilized a community-based participatory research orientation, or CBPR. Bush et al. (2017) assert that “CBPR is an approach in which researchers undertake research in partnership with those affected by the issue being studied, for the purpose of taking action or effecting social change; it can also incorporate those who will use the results to change practice and inform policy. CBPR is research with communities rather than research on or about communities” [22,23]. Therefore, by utilizing this research orientation, the research team was able to ensure that the unique needs identified by the participating communities and schools would be the central focus [22,23]. These participating school districts identified the need for a trauma-informed, movement-based intervention to mitigate the ongoing mental health and suicide crisis in their communities. Thus, the idea of piloting a school-based, trauma-informed yoga intervention was born.

There is a significant lack of research combining the topics of rurality and remotely delivered trauma-informed yoga interventions. However, there is existing research that supports the efficacy of remotely delivered yoga interventions, alongside research on yoga in rural contexts. Previous studies indicate that there is a clear difference between participation in yoga activities in urban settings as compared with rural settings; this is likely attributed to the lack of access to qualified instructors in more rural contexts [24–26]. Further, since the onset of COVID-19, remotely delivered yoga interventions have risen in popularity due to the lockdown associated with the pandemic. A 2024 meta-analysis of randomized control trials with remote and online interventions during the pandemic showed moderate to large effects on both anxiety and depression, respectively, among children, adolescents, and young adults [27]. Recent studies also indicate that this delivery method has been successful in promoting mental and physical well-being across adult populations [28,29]. Our study draws on this previous research and seeks to address the gap in the literature by comparing both an in-person and remotely delivered, trauma-informed intervention for both adolescent and adult populations.

While there is a plethora of research supporting mental, physical, and cognitive benefits associated with yoga practice, a trauma-informed yoga practice differs from that of a traditional one [6,25]. Specifically, for trauma survivors, the element of “somatic experiencing” is critical to a yoga practice to move through one’s trauma [30]. According to Levine et al. (2018), “somatic is a reference to the Greek soma, signifying something that is of the living body. Experiencing is a verb, alluding to interventions that access the phenomenological level of being and are focused on the present moment” [31] (p. 620). Somatic Experiencing (SE) focuses on bodily sensations as a therapeutic approach to resolving trauma and associated symptoms [31]. Trauma-informed yoga provides instruction and experience that supports individuals who may respond to triggers while also striving to prevent re-traumatization [10,25]. According to Spinazzola et al. (2018), the effects of trauma can severely impact both psychosocial functioning and neurodevelopment in youth [32]. Trauma also disrupts the vagus nerve, which is crucial for regulating autonomic functions and emotional responses, contributing to heightened arousal and emotional dysregulation [32]. However, regular physical activity, including yoga, has been shown to mitigate symptoms of PTSD and improve cognitive function; specifically, for individuals who have experienced trauma, consistently higher levels of cortisol can have a detrimental effect on the hippocampus, thus negatively impacting memory and stress regulation [33]. Exercise promotes neuroplasticity and neurogenesis, counteracting the detrimental effects of chronic stress on the brain [34]. Physical movement also enhances mood regulation and self-efficacy, which is therefore essential for trauma recovery [35].

Integrating somatic experiencing principles into yoga practice in an educational environment offers a promising approach for trauma survivors to heal and regain control over their bodies and minds. By addressing the physiological impacts of trauma and leveraging the benefits of movement and exercise, students can experience improvements in their well-being and quality of life. Although prolonged stress can negatively impact the entirety of the body, developmentally appropriate opportunities in an educational setting—like a trauma-informed yoga practice—can help improve the stress response’s flexibility and capability to support building resiliency [36]. Offering these kinds of opportunities is an important part of the process to counteract the negative effects of trauma and adverse childhood experiences since, according to Smyth and Pollack (2020), “early life stress is consistently associated with altered functioning of the hypothalamic pituitary adrenal (HPA) axis and autonomic nervous system” [7] (p. 3). Research by Chiang, Lam, Chen, and Miller (2022) also found that “psychological stress during childhood and adolescence increases risk of health problems across the life course” [3] (p. 27). From surviving to thriving, supporting students in their mental health journeys exhibits great promise through trauma-informed interventions, such as the one utilized in this study, which in turn provide positive childhood experiences (PCEs) and ultimately health outcomes of positive experiences (HOPEs) [6].

### 1.5. Purpose of the Study

As described previously, there is a plethora of research supporting both the negative outcomes of childhood adversity and the need for movement-based therapies; however, there is a dearth of research investigating novel, school-based programming designed to mitigate adverse childhood experiences, especially in rural settings [4,8,25,30,35]. Thus, this pilot study was designed to address this major gap in the literature and builds on 4 years of previous iterations of this project, which piloted different modalities of the intervention to both high school students and educators [37–42]. In this culminating, final year, the aim of this project was to determine which intervention modality was more effective with students across two different school districts with varying degrees of rurality (i.e., if the intervention was delivered in-person vs. remotely over Zoom). A secondary aim of this study was to determine the efficacy of a remotely delivered, concurrent intervention for educators across both school districts. Comparisons of mental and physical health outcomes were performed by the research team using cohort data drawn from participants’ physiological data and survey results. The present study sought to explore the effects of trauma-informed yoga intervention on rural youth and their teachers.

## 2. Materials and Methods

### 2.1. Participants

This pilot study was conducted in rural southwestern Montana across two different school districts. The first school was in a rural town with about 8500 residents, while the second was in a very rural, geographically remote location with fewer than 800 year-round residents. For the academic year in which this study was conducted, the district in the larger town had a total of 1255 students, with the high school enrolling over 400 students. In the remote school district, the entire district had fewer than 150 students, with the high school comprising roughly one-third of all students [43]. Participants’ ages ranged from 15 to 17 years of age.

### 2.2. Timing

This study was held during the first 6 weeks of the third academic quarter (end of January through mid-March 2023). This time period was identified by school district administrators as one that is historically difficult for students, as attendance rates tend to drop and reported cases of mental crisis increase. Additionally, this is a challenging time for educators, as negative student behaviors and the aforementioned absenteeism typically rise during this time.

### 2.3. Intervention Design

As previously stated, our community-based participatory research orientation allowed the research team to co-create our intervention design alongside our school and community partners. The resulting intervention design was composed of three primary groups of interest: (1) a face-to-face intervention group for students in the larger rural high school; (2) a synchronous, remotely delivered intervention group for the very rural geographically isolated high school; and (3) a synchronous, remotely delivered intervention group for educators across both districts. Across all three groups, 83 students and educators volunteered to participate in our study. Educators were offered the option to receive their intervention before or after school, depending on their schedules. For students, the intervention took place in their regularly scheduled physical education class; for all experimental groups, the intervention took place during the same 6-week timeframe. Sessions for all participants were held twice weekly for the six weeks of the intervention, and all sessions were 45 min long, regardless of the delivery modality. All intervention sessions were led by two certified trauma-informed yoga instructors (one instructor delivering the intervention in person and the other leading the sessions remotely). Additional characteristics of the schools included variability in their school calendar: the face-to-face student group’s school district is held on a 5-day school week, whereas the more rural school operates on a 4-day school week. Participation by all enrollees was completely voluntary, and participation incentives were offered; high school students received a wearable fitness tracker upon the completion of the study, and educators were offered an Amazon gift card upon the conclusion of their participation.

### 2.4. Aim One

In total, 18 students were enrolled from the larger rural district, and 22 were enrolled from the remote district. Student ages ranged from 15 to 17. While racial data were not collected, there were 9 females enrolled from the larger high school and 9 males, and there were 11 females and 11 males from the smaller high school.

### 2.5. Aim Two

Forty-three educators volunteered to participate across both districts; given that they all received the same intervention modality, they were not separated into distinct cohorts in the experimental design. In terms of the participating educators, 39 females and 4 males were enrolled; more educators were initially enrolled, but they did not complete the study due to attrition. Educator ages ranged from 27 to 77.

**Figure 1.**
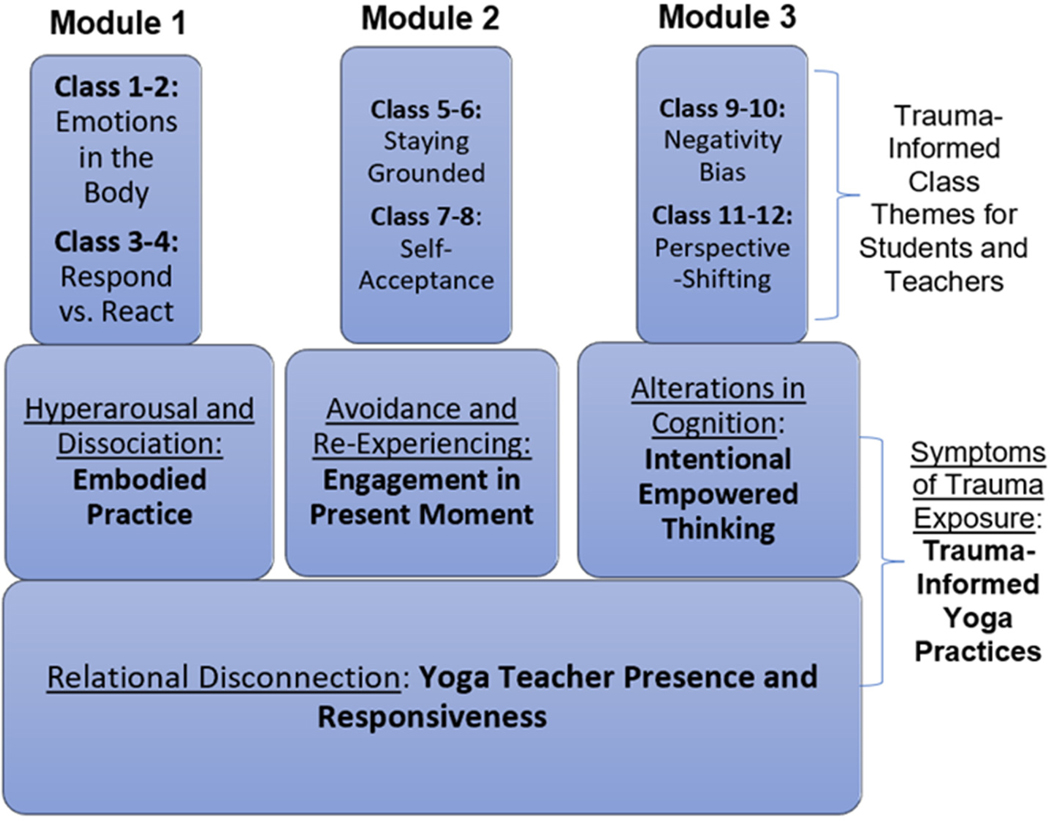
Intervention design, adapted from Cook-Cottone (2020) [35].

In line with established research findings, Figure 1 below illustrates our trauma-informed intervention framework, as adapted from Cook-Cottone [35].

As outlined above, each week of the intervention was intended to explore various themes related to the embodiment of trauma in an attempt to reestablish feelings of agency and positivity. Within each module, specific movement sequences were taught to participants to address specific symptoms of traumatic exposure, and the entire intervention was grounded in addressing the overall relational disconnect that occurs among trauma survivors. These intentional practices began with instructors helping participants set an intention, or goal, for their practice (based on the theme of the class/week/module), and participants were asked to respond to journal prompts related to these intentions at the conclusion of each session as an opportunity for reflection.

### 2.6. Survey Measures

#### 2.6.1. Adverse Childhood Experiences

Adverse Childhood Experiences for Adults (ACE-Adults; [44]). The ACE-Adults is a 10-item measure that assesses the presence of adverse childhood experiences (ACEs). We asked educators to retrospectively report the presence (coded 1) or absence (coded 0) of each adverse childhood experience (abuse or maltreatment, neglect, household dysfunction) before the age of 18 years old; we also presented an additional question asking whether they felt these events impacted their health. We used the total summed score across all items in this study with higher scores indicating a greater number of ACEs educators experienced before age 18 years old. Past research has shown that the retrospective reports of ACEs are valid, with few false positives [44].

#### 2.6.2. Center for Youth Wellness Adverse Childhood Experiences Questionnaire (CYW ACE-Q Teen Version [45])

The CYW ACE-Q Teen Version is a 2-section self-report questionnaire that assesses the number of adverse childhood experiences youth have been exposed to since birth [46]. We only presented students with the first section. Specifically, we asked students to report how many specific statements applied to them from the first section. Section 1 included events from the original ten ACEs identified (e.g., Someone pushed, grabbed, slapped, or threw something at you OR you were hit so hard that you were injured or had marks; your parents were separated or divorced). We did not include Section 2 of the instrument, which involved nine supplementary ACEs (e.g., you have been in foster care; you have lived with a parent or guardian who died). We used the total summed score across the first section (ranging from 0 to 10), with higher scores indicating a greater number of ACEs youth experienced since birth.

#### 2.6.3. Mental and Behavioral Health

Generalized Anxiety Disorder—7 (GAD-7-Adult and GAD-7-Child; [46–48]). The GAD-7-Adult and GAD-7-Child are 7-item measures that assess the severity of generalized anxiety disorder symptoms in adults and youth, respectively. We asked educators and students to report how bothered they were by each symptom over the past two weeks using a 4-point Likert-type scale consisting of 0 (not at all), 1 (several days), 2 (more than half the days), and 3 (nearly every day). We used mean scores across all items, with higher scores indicating greater severity of anxiety disorder symptoms for educators and students. Previous research showed the GAD-7-Adult has excellent internal consistency (e.g., α = 0.92), construct validity (all items loaded onto a single factor), and convergent validity with established measures of anxiety disorder symptoms (e.g., *r* = 0.72 with Beck Anxiety Inventory) [46–48]. The GAD-7-Adult and GAD-7-Child showed good to excellent internal consistency at pre-intervention (educators: α = 0.91; students: a = 0.90) and post-intervention (educators: a = 0.93; students: α = 0.81).

#### 2.6.4. Patient Health Questionnaire—9 and Patient Health Questionnaire—A (PHQ-9 and PHQ-A; [49,50])

The PHQ-9 and PHQ-A are 9-item measures assessing the frequency of depressive symptoms in adults or youth. We asked educators and students how often they experienced each problem over the past two weeks using a 4-point Likert-type scale consisting of 0 (not at all), 1 (several days), 2 (more than half the days), and 3 (nearly every day). We used the mean score across all items of the PHQ-9 and PHQ-A, with higher scores indicating a greater frequency of depressive symptoms in the past two weeks for educators or students, respectively. Research has shown that both the PHQ-9 and PHQ-A are reliable and valid measures of depressive severity, with good internal consistency estimates (e.g., α = 0.89 for adults) and excellent sensitivity to distinguishing between individuals with and without a depressive disorder [49,50]. The PHQ-9 and PHQ-A showed good to excellent internal consistency at pre-intervention (educators: α = 0.87; students: α = 0.91) and post-intervention (educators: α = 0.89; students: α = 0.91).

#### 2.6.5. PTSD Checklist for DSM-5: Adult Version (PCL-5-Adults; [51])

The PCL-5-Adults is a 20-item self-report measure that assesses DSM-5 PTSD symptoms in adults. One school district was not comfortable with their students completing an adolescent version of the PCL-5, and thus the adolescent version was eliminated from the survey for both school districts. We asked educators to rate how much each symptom bothered them over the past month using a 5-point Likert-type scale consisting of 0 (not at all), 1 (a little bit), 2 (moderately, 3 (quite a bit), and 4 (extremely). We used the mean score across all items, with higher scores indicating greater severity of PTSD symptoms in the past month for educators. Previous research has shown the PCL-5 has excellent internal consistency (α = 0.94) and convergent validity with other established measures of PTSD symptoms and distress (e.g., *r* = 0.74 and *r* = 0.85) [51]. The PCL-5 showed excellent internal consistency at pre-intervention (educators: α = 0.95) and post-intervention (educators: α = 0.93).

#### 2.6.6. Patient-Reported Outcomes Measurement Information System—Sleep Disturbance Short Form for Adults and Children Ages 11–17 (PROMIS SD-SF—Adult/PROMIS SD-SF—Child; [52])

The PROMIS SD-SF—Adult and PROMIS SD-SF—Child are identical 8-item measures that assess sleep disturbances in adults or 11–17-year-old youth, including difficulties in sleep onset, sleep quality, and sleep continuity. We asked educators and students to indicate how much they had been bothered by symptoms of sleep disturbance over the past 7 days on a 5-point scale consisting of 1 (not at all), 2 (a little bit), 3 (somewhat), 4 (quite a bit), and 5 (very much) for four items (e.g., “I had difficulty falling asleep”); how often symptoms occurred on a 5-point scale consisting of 1 (never), 2 (rarely), 3 (sometimes), 4 (often), and 5 (always) for three items (e.g., “I had trouble sleeping”); and the quality of their sleep on a 5-point scale consisting of 1 (very poor), 2 (poor), 3 (fair), 4 (good), and 5 (very good) for four items. For the items that were reverse-coded, we transformed the scale so that the higher number corresponded to more sleep disturbance. We used mean scores across all items, with higher scores indicating greater sleep disturbance in educators or students. Two studies of the initial development and validation of the two PROMIS SD-SF short forms with both adults and youth have shown excellent internal consistency estimates (marginal proxy A < 0.90) and convergent validity with other established measures of sleep problems (e.g., *r* = 0.83 with sleep quality in adults and *r* = 0.49 with sleep habit survey in youth) [52]. The PROMIS SD-SF—Adult and PROMIS SD-SF—Child showed excellent internal consistency at pre-intervention (educators: a = 0.96; students: a = 0.95) and post-intervention (educators: a = 0.93; students: a = 0.96).

#### 2.6.7. Connor–Davidson Resilience Scale (CD-RISC; [53])

The CD-RISC is a 25-item self-report measure that assesses an individual’s general resilience. We asked educators and students to rate how often each statement on the CD-RISC was true of them over the past month using a 5-point scale consisting of 0 (not true at all), 1 (rarely true), 2 (sometimes true), 3 (often true), and 4 (true nearly all the time). We used the mean score across all items, with higher scores indicating greater resilience in educators or students. Research has shown the CD-RISC to have good internal consistency (a < 0.89) and convergent validity with measures of hardiness (*r* = 0.83) and perceived stress vulnerability (*r* = −0.76) [53]. The CD-RISC showed good to excellent internal consistency at pre-intervention (educators: a = 0.86; students: a = 0.90) and post-intervention (educators: a = 0.88; students: a = 0.90).

#### 2.6.8. Educator Career Satisfaction Professional Quality of Life Index (ProQOL; [54])

The ProQOL is a 30-item measure that assesses an individual’s career satisfaction and professional burnout across three domains: compassion satisfaction (10 items), burnout (10 items), and secondary traumatic stress (10 items). We asked educators how often they experienced an item on a 5-point Likert-type scale consisting of 1 (never), 2 (rare), 3 (sometimes), 4 (often), and 5 (very often). We used the mean score for all items from each domain, with higher scores indicating greater compassion satisfaction, burnout, and secondary traumatic stress. Previous research has shown acceptable to good internal consistency for each subscale (a = 0.88 for compassion satisfaction, a = 0.75 for burnout, a = 0.81 for secondary traumatic stress) and construct validity, demonstrating that each subscale is distinct [55]. The ProQOL showed excellent internal consistency for compression satisfaction at pre-intervention (educators: a = 0.95) and post-intervention (educators: a = 0.95), acceptable internal consistency for burnout at pre-intervention (educators: a = 0.86) and post-intervention (educators: a = 0.86), and acceptable internal consistency for secondary traumatic stress at pre-intervention (educators: a = 0.81) and post-intervention (educators: a = 0.78).

#### 2.6.9. Educator Sense of Self-Efficacy—Short Form (TSSE-SF; [55])

The TSSE-SF is a 12-item measure that assesses educators’ sense of self-efficacy and agency in their careers across three domains of efficacy: student engagement, instructional strategies, and classroom management. We asked educators to rate how much they could do for each item (e.g., “How much can you do to motivate students who show low interest in schoolwork?”; “How much can you do to control disruptive behavior in the classroom?”) on a 9-point Likert scale, ranging from a 1 (not at all) to 9 (a great deal). We used the mean score for all items, with higher scores indicating a greater sense of self-efficacy as an educator. Past research has shown good to excellent internal consistency for each domain (Cronbach’s alpha ranging from 0.81 to 0.91) and construct validity with items loading onto the three domains of self-efficacy [56]. The TSSE-SF showed acceptable to good internal consistency for the efficacy of student engagement at pre-intervention (educators: a = 0.82) and post-intervention (educators: a = 0.78), good internal consistency for the efficacy of instructional strategies at pre-intervention (educators: a = 0.81) and post-intervention (educators: a = 0.81), and good to excellent internal consistency for the efficacy of classroom management at pre-intervention (educators: a = 0.88) and post-intervention (educators: a = 0.94).

### 2.7. Survey Data Analytic Plan

Analyses were performed using R Statistical Software (v4.3.3; [56]). Reliability and validity measures were calculated using the psych R package (v2.4.3) [57]. Linear mixed models were calculated using the lme4 R package (v1.1.35.2) [58], Type III ANOVA tests for linear mixed models were performed with the car R package (v3.1.2) [59], statistics and model marginal mean effects were calculated and visualized using the ggeffects R package (v1.5.1) [60], and assumptions were assessed by visual examination of the diagnostic plots created by the performance R package (v0.12.3) [61].

All response variables were fit to a linear mixed model accounting for the random effects from the repeated measurements on educators with the pre- and post-intervention survey design. In initial models, we tested for an interaction between the ACE score, the timing of the survey, and the gender of the educator. If there was little evidence for the three-way interaction (*p*-values > 0.05), all two-way interactions were tested also accounting for the age and number of sessions attended for each educator, adjusting for the random effects from the repeated measurements design. The school district was not included as a fixed effect as the educators received the material in the same way.

The response variables were created based on the design of the specific validated survey instrument’s specifications with the exception of using the mean of the items instead of the sum of the items. This helps ensure that the residuals from the model are normally distributed and on the same scale as the survey items for the particular instrument. As all survey items were forced responses, there should be no missing values.

#### 2.7.1. Educator Survey Measures

The main effects of the adverse childhood experience (ACE) score; the timing of the survey (pre- or post-intervention); gender; the number of sessions attended, age; and the interaction between the ACE score, timing, and gender were included in the model as fixed effects. Model reduction allowed all fixed effect terms to be removed from the model (except for the timing of the survey) if there was little evidence that the term was important to the model (*p*-value > 0.05).

#### 2.7.2. Student Survey Measures

The main effects of students’ ACE scores; timing of the survey (pre- or post-intervention); gender; the number of sessions attended; age; school district; and up to and including the four-way interaction between the ACE score, timing, gender, and district were included in the model as fixed effects. Model reduction allowed all fixed effect terms to be removed from the model except for the timing of the survey if there was little evidence that the term was important to the model (*p*-value > 0.05).

As with the educator survey measures, all response variables were fit to a linear mixed model accounting for the random effects from the repeated measurements on students with the pre- and post-intervention survey design. In the initial models, we tested for an interaction between the ACE score, timing of the survey, gender, and school district for the students. If there was little evidence for the four-way interaction (*p*-values > 0.05), all three-way interactions were tested, also accounting for the age and number of sessions attended for each student, adjusting for the random effects from the repeated-measurement design. Terms or interaction terms were removed one at a time until either only the timing of the survey was left, or all terms (except for perhaps timing) had a *p*-value < 0.05.

Again, just as with the educators’ surveys, the response variables were created based on the design of the specific validated survey instrument’s specifications with the exception of using the mean of the items instead of the sum of the items; all survey items were a forced response; therefore, there should be no missing values.

### 2.8. Psychophysiological Measures and Data Analytic Plan

#### 2.8.1. Heart Rate Variability

Resting heart rate patterns were collected in the classroom for students (3 min) and educators (2 min) using photoplethysmography (PPG) sensors placed on the middle and index fingers of their nondominant hand. PPG sensors transmitted signals wirelessly to the Biopac MP-160 BioNomadix Wireless system (Biopac Systems Inc., Goleta, CA, USA) and recorded digitally to the accompanying Acknowledge 5.0 software (Biopac, USA) on a Lenovo ThinkPad Laptop, 11th Gen Intel ^®^ Core^™^ i5–1135G7, 2.40 GHz, 8 GB RAM using a Windows 10 operating system. Data collection was performed in a classroom setting with five participants sitting at desks facing away from other students and educators to reduce distraction. In addition, all five participants wore protective earmuffs during data collection to minimize external noise. PPG data were electronically stored on a password-protected laptop and transferred to an encrypted server hosted by Montana State University (Microsoft OneDrive Business). All data were de-identified before processing and statistical analysis.

Student and educator PPG inter-beat interval (IBI) data for pre- and post-intervention sessions were processed using Mindware HRV Analysis 3.2 software (Mindware Technologies Inc., 2022, Gahanna, OH, USA) to derive the mean heart rate (HR), high-frequency heart rate variability (HF-HRV), and root mean squared of successive differences (RMSSD). An automatic artifact detection algorithm was used to detect potential artifacts in the IBI data [62]. Flagged data were visually inspected for artifacts (e.g., identify missed or extra beats) and manually corrected. We created 60 s time series epochs from the artifact-free IBIs across the resting baseline for students and educators using an interpolation algorithm, linearly detrended to remove non-stationary data, and used a Hamming window [63]. We then subjected each 60 s epoch to a power spectral analysis using a fast Fourier transform to produce absolute power (or variance) distribution for the HF-HRV band within that epoch. We used age-adjusted high-frequency bands as outlined in Shader et al. [64], where the lower bound value ranged from 0.15 [age 17] to 0.20 [age 14], and the upper bound value was set at 0.50 for all ages. HF-HRV values for each epoch were natural-log-transformed (ln) due to severe positive skew. The mean HR, HF-HRV, and RMSSD from each 60 s epoch were averaged across the resting baseline to derive a single mean HR, HF-HRV, and RMSSD value for the student and educator resting baseline at pre- and post-intervention. We conducted three paired-sample *t*-tests to evaluate the pre- to post-intervention change in mean HR, HF-HRV, and RMSSD for both students and educators.

Finally, while all students provided HRV data, not all educators were able to do so. As mentioned in the study context section of this manuscript, weather often creates a significant barrier to access in rural Montana; the day the research team was to gather HRV data from educators in the remote location, a blizzard hit, so the research team had to leave the study site early to return home safely. Therefore, our HRV data for educators only represent educators from the larger school district (where the weather did not create an interruption in data collection).

#### 2.8.2. Cortisol

Study participants were given salivary cortisol testing at baseline (beginning of week 1, prior to the intervention beginning) and at the intervention’s conclusion (following the final session at the end of week 6). To address the variability of cortisol levels throughout the day, collection times were kept consistent between both high school groups and the educator group. Salivary cortisol was collected on-site during the physical health periods of the high school students (roughly the same time mid-morning across both districts) and in the afternoons for educators (also around the same time in mid/late afternoon).

Participants provided a saliva sample to PI, which was de-identified (using a code key system) before analysis by the Center for American Indian and Rural Health Equity’s Translational Biomarkers Core Lab at Montana State University. Testing used the Abcam (ab154996) cortisol in vitro competitive ELISA (enzyme-linked immunosorbent assay) kit designed for accurate quantitative measurement of cortisol in saliva (sensitivity 0.12 ng/mL). De-identified cortisol data were returned to the PI for re-identification using the code key, and comparisons using paired *t*-tests assessed trends to determine intervention impacts. These paired-sample *t*-tests assessed differences in means at time 3 vs. time 1, overall and by gender. The mean accuracy of salivary cortisol testing utilizing these assays is greater than 90%.

### 2.9. Qualitative Feedback

All participants were asked to provide feedback to open-ended questions at the conclusion of the intervention. The questions were asked at the end of the survey. Sample questions included “How did the yoga classes make you feel emotionally and mentally (feelings, stress, anxiety, etc.)?”, “How did the yoga classes make you feel physically (in your body)?”, and “Did yoga help you cope with the stress caused by COVID-19 or other life circumstances? If so, how?” Although we did not conduct a formal qualitative analysis of the open-ended responses, sample participant responses are shared to provide insights that would not be otherwise captured by our survey data.

## 3. Results

### 3.1. Survey Measures

#### 3.1.1. Students

Broadly, there were many positive changes in mental health measures for students across both districts, regardless of the intervention modality. Descriptive statistics across both districts indicated improvement across most survey measures; average depression (PHQ-A) scores were reduced by a mean of 2.8 points, anxiety (GAD-7) scores were reduced by a mean of 1.5 points, and sleep disturbances (PROMIS) were reduced by a mean of 0.433 points. However, resilience (CD-RISC) scores increased by an average of 0.4 points across both districts. The only survey measure that showed evidence of a difference in mean values for pre-intervention scores as well as post-intervention scores was the PHQ-A (see Table 1). A linear model testing for differences between mean scores between districts, assuming equal variances showed very strong evidence of a difference between districts for the pre-intervention scores (*t*-value = −2.844, df = 39, *p*-value = 0.007), and strong evidence of a difference in mean scores between districts for the post-intervention scores (*t*-value = −2.121, df = 39, *p*-value = 0.0413).

Statistical analyses accounting for the repeated measurements before and after the intervention and including interactions between gender and adverse childhood experience (ACE) scores, for each of these measures, are described in detail below. Student age was important only for the PROMIS model, and the number of intervention sessions they attended did not statistically significantly impact any of the scores for students and was therefore not included in any of the final models. Model assumptions were assessed by a visual examination of the diagnostic plots [Supplementary Materials]. There was little evidence against the linearity, constant variance, influential observations, normality of residuals, and normality of random effects for all of the following models, with the exception of the PROMIS scale. Here, there was some evidence against the normality of residual assumption, with a few residuals with extreme values; however, these observations were not influential to the model.

#### 3.1.2. Depression: PHQ-A

The final model for PHQ-A included a three-way interaction between students’ ACE scores, their district, and gender (*t*-value = 2.274, df = 32.58, *p*-value = 0.0297), and all nested two-way and main effects for ACE, district, and gender, as well as the timing of the survey (pre- or post-intervention) (*t*-value = −2.968, df = 37.43, *p*-value = 0.0052). It also included the repeated measures for students’ random effect. The results indicated that for an increase of 1 point in ACE scores, females in the less rural (in-person intervention) school district (who received the in-person intervention), on average, had an increase of 0.069 points on the Likert scale in their PHQ-A score. Males in the same district had an increased depression score of 0.025 points, on average, as their ACE score increased. Similarly, for an increase of 1 point in the ACE scale, females from the very rural, remote district (virtual intervention), on average, had an increase of 0.047 points in the PHQ-A scale, and males had an increase of 0.254 points. Thus, male students from the very rural, remote (virtual intervention) school district showed the highest increase in PHQ-A scores, on average, after accounting for the other variables in the model, and females from the less rural school district (in-person intervention) had the smallest increase in PHQ-A scores.

#### 3.1.3. Anxiety: GAD-7

For the GAD-7 instrument, the final model included the two-way interaction between students’ ACE scores and the timing of the survey (pre- or post-intervention) (*t*-value = −2.118, df = 33.47, *p*-value = 0.0417) and their main effect terms, gender as a fixed effect (*t*-value = 2.882, df = 34.83, *p*-value = 0.0067), and the repeated measures for students’ random effect. Findings showed that as students’ ACE scores increased by 1, on average, their anxiety scores decreased by 0.086 in the post-intervention survey. Additionally, females had a higher GAD-7 score before the intervention as compared to males, but there was no evidence that the intervention resulted in different changes in GAD-7 scores for males and females in post-surveys. Neither the number of sessions attended nor the school district had any impact on the scores for this instrument.

The GAD-7 scores were 0.62 points lower in the post-intervention survey compared to the pre-intervention survey for those students with an ACE score of 9, and for students with an ACE score of 1, the GAD-7 scores were 0.072 points higher in the post-intervention survey. For an *ACE* score of 9, the estimated values were $\overset{ˆ}{\beta }$ = −0.62, se = 0.216, df = 35, *t*-value = −2.873, and *p*-value = 0.0069; for an ACE score of 1, they were $\overset{ˆ}{\beta }$ = 0.072, se = 0.185, df = 36, *t*-value = 0.39, and *p*-value = 0.6986.

#### 3.1.4. Sleep Quality: PROMIS

In terms of sleep quality, the intervention had an impact on the scores for the PROMIS sleep disturbance scale (*t*-value = −3.188, df = 36.5, *p*-value = 0.0029). The final model for the PROMIS sleep disturbance scale included the timing of the survey, the age of the student, the two-way interaction between the ACE score and district, and the repeated measures for random effects. For an increase of 1 point in adverse childhood events, students from the larger district, on average, had an increase of 0.01 points on the PROMIS sleep disturbance scale, whereas students from the very rural (virtual intervention) district, on average, had an increase of 0.252 points for the PROMIS sleep disturbance scale. Therefore, students from the more rural district showed the highest increase in PROMIS sleep disturbance scores, on average, indicating more sleep difficulties; after accounting for the other variables in the model, students from the larger, less rural district (in-person intervention) had the lowest increase in those scores. Regardless of district, on average, students showed a decrease in PROMIS scores in the post-intervention survey, after adjusting for their age, ACE score, district, and the interaction between ACE score and district, which indicates an improvement in sleep quality.

#### 3.1.5. Resilience: CD-RISC

This intervention also had no statistically significant impact on scores for the CD-RISC (*t*-value = 0.571, df = 35.37, *p*-value = 0.57191) after accounting for the other terms in the model. The final model for CD-RISC included a two-way interaction between students’ ACE scores and gender. The gender of the student (*t*-value = 2.457, df = 35.43, *p*-value = 0.01904) and their main effect terms, the timing of the survey (pre- or post-interaction) as a fixed effect, and the repeated measures for students’ random effect were considered. As students’ ACE scores increased by 1, on average, their CD-RISC scores increased by 0.0189 for females ($\overset{ˆ}{\beta }$ = 0.189, se = 0.08) and decreased by 0.145 for males ($\overset{ˆ}{\beta }$ = −0.145, se = 0.06) from pre- to post-intervention. This indicates that for females, for each additional point on the ACE scale, resilience scores increased, whereas for males, their resilience decreased as their ACE scores increased. Table 2 includes the results from the final models for the scales, indicating significant interactions found between pre- and post-intervention measures.

#### 3.1.6. Educators

The intervention resulted in changes in the scores (pre- to post-intervention) of all mental health survey measures for educators, including the CD-RISC, GAD-7, PCL-5, PHQ-9, and Professional Quality of Life (ProQOL) secondary traumatic stress scales. Descriptive statistics indicated overall very positive outcomes. Average depression (PHQ-9) scores were reduced by 2.5 points, anxiety (GAD-7) scores were reduced by 2.4 points, and trauma symptoms (PCL-5) were reduced by 7.7 points. Sleep disturbances (PROMIS) were also reduced by 0.5 points, indicating an improvement, and contrary to the student groups, average educator resilience (CD-RISC) scores increased by 0.6 points.

In terms of career satisfaction measures, descriptive results were mixed. Overall, educators reported reduced self-efficacy by 1.3 points, and mixed improvements were observed in the subscales of the Educator Sense of Self-Efficacy (TSSE) instrument; student engagement scores decreased by 0.9 points, instruction strategies increased by 0.1 points, and classroom management was reduced by 0.5 points. Lastly, the Professional Quality of Life Index (ProQOL) exhibited mixed improvements in the subscales; while compassion satisfaction was reduced by 0.7 points, on the positive end, burnout was reduced by 0.6 points, and compassion fatigue was reduced by 3 points.

Statistical analyses, including interactions between the number of sessions attended, gender, and adverse childhood experience (ACE) scores, for each of these measures, are described in detail below. Model assumptions were assessed by visual examination of the diagnostic plots. There was little evidence against the linearity, constant variance, influential observations, normality of residuals, and normality of random effects for all of the models, with the exception of the PCL-5 scale and the professional “burnout” subscale. Here, there was some evidence against the normality of residual assumption, with a few residuals with extreme values; however, these observations were not influential to their respective models.

#### 3.1.7. Depression: PHQ-9

The final model for the PHQ-9 included a two-way interaction between the timing of the survey (pre- or post-intervention), gender (*t*-value = −2.219, df = 44.10, *p*-value = 0.0317), number of sessions attended as a fixed effect (*t*-value = −2.7, df = 44.23, *p*-value = 0.0098), and the repeated measures for educators’ random effect. For the pre-intervention surveys, females self-reported higher PHQ-9 scores than males, on average, but they reported a mean of 0.559 points lower on the average PHQ-9 scores for the post-intervention survey. Male participants, however, showed a small increase in the PHQ-9 scores in the post-intervention survey compared to the pre-intervention survey, but it is possible that this increase is due to random chance, as the timing variable by itself was not important to the model (*t*-value = 0.973, df = 44.81, *p*-value = 0.3360). The number of sessions attended was also important to the model, and with a negative coefficient, this indicated that as the number of sessions attended by adult participants increased by 1, on average, PHQ-9 scores decreased by 0.058 (after accounting for the other fixed effects and random effects in the model).

#### 3.1.8. Anxiety: GAD-7

For the GAD-7 survey instrument, the final model only contained the time variable, and this variable did have a significant *p*-value (*t*-value = −3.462, df = 37.37, *p*-value = 0.0014), indicating that there was evidence of a difference in GAD-7 scores between the pre-intervention and the post-intervention surveys, after accounting for the repeated measurements of the educators. As the coefficient of the survey’s timing was negative, this indicated that, on average, the GAD-7 score was 0.3529 points lower in the post-intervention survey than in the pre-intervention survey. Variables of participant age, gender, the ACE score, and the number of sessions attended did not impact the model.

#### 3.1.9. Sleep Quality: PROMIS

The final model for the PROMIS sleep disturbance scale included the ACE score, the timing of the survey, the number of sessions attended, and the repeated measures for random effects. There was very strong evidence that the timing of the survey resulted in a change in scores from the pre- to post-intervention surveys, indicating an improvement in sleep quality ($\overset{ˆ}{\beta }$ = −0.469, se = 0.125, *t*-value = −3.75, df = 39.99, *p*-value = 0.0006). The educators’ ACE scores did impact the effectiveness of the intervention. As the ACE score increased by 1, on average, the PROMIS sleep disturbance score increased by 0.123, after accounting for the other variables, regardless of the timing of the survey and the number of sessions attended, which indicates decreased sleep quality for these participants ($\overset{ˆ}{\beta }$ = 0.123, se = 0.055, *t*-value = 2.236, df = 41.7, *p*-value = 0.0308). Lastly, when more sessions were attended, a greater decrease was observed in the average PROMIS sleep disturbance scores, which is indicative of improved sleep quality. For each additional session attended, on average, the PROMIS scores decreased by 0.0797, after accounting for the ACE score, the timing of the survey, and the random effects ($\overset{ˆ}{\beta }$ = −0.0797, se = 0.038, *t*-value = −2.069, df = 45.9, *p*-value = 0.0442).

#### 3.1.10. Resilience: CD-RISC

The final model for CD-RISC included a two-way interaction between the participants’ ACE score and the timing of the survey (pre- or post-intervention) (*t*-value = −2.809, df = 38.89, *p*-value = 0.0077), their main effect terms, gender as a fixed effect (*t*-value = −2.488, df = 45.74, *p*-value = 0.0166), and the repeated measures for educators’ random effect. As the participants’ ACE score increased by 1, on average, the CD-RISC score decreased by 0.082 on the post-intervention survey compared to the pre-intervention survey. This is indicative that the intervention resulted in a greater decrease in resilience scores for those who had higher ACE scores. Additionally, females had a lower CD-RISC score to begin with compared to males, but there was no evidence that the intervention resulted in different changes in CD-RISC scores for males and females. The number of sessions attended also did not seem to make a difference in scores for this instrument.

#### 3.1.11. Trauma Symptoms: PCL-5

As with the GAD-7 survey instrument, the final model for PCL-5 also just contained the timing of the survey as a variable, along with the repeated measures for random effect with a small *p*-value (*t*-value = −4.346, df = 38.68, *p*-value < 0.0001); this similarly indicates that there was evidence of a difference in PCL-5 scores after the intervention when compared to the pre-intervention scores. On average, the PCL-5 scores were between “not at all” and “a little bit” on the pre-intervention survey, but there was still an important reduction in the scores on the post-intervention survey regardless of age, gender, ACE score, and number of sessions attended.

#### 3.1.12. Professional Quality of Life Index: ProQOL

For the Professional Quality of Life survey instrument, there were three subscales; these subscales were analyzed separately. They included compassion satisfaction, burnout, and secondary traumatic stress.

For compassion satisfaction, the final model included participants’ ACE score, the timing of the survey, and the number of sessions attended, as well as the repeated measures for random effects. There was little evidence that the intervention changed the scores for this subscale ($\overset{ˆ}{\beta }$ = −0.101, *t*-value = −1.126, df = 36.88, *p*-value = 0.2673), but there was a slight decrease in scores from the pre- to post-intervention surveys.

The educators’ ACE score did impact the effectiveness of the intervention, however; as ACE scores increased by one, on average, the compassion satisfaction score increased by 0.111, after accounting for the other variables, regardless of age and gender ($\overset{ˆ}{\beta }$ = 0.111, *t*-value = 2.689, df = 38.75, *p*-value = 0.011).

Lastly, the number of sessions attended also had a positive impact on the compassion satisfaction scores. For each additional session attended, on average, compassion satisfaction scores increased by 0.078, after accounting for the ACE score, the timing of the survey, and the random effects ($\overset{ˆ}{\beta }$ = 0.078, *t*-value = 2.706, df = 42.7, *p*-value = 0.0098).

On the professional “burnout” subscale, none of the variables measured seemed to explain the average score for these items. In particular, the timing variable was not important to the model ($\overset{ˆ}{\beta }$ = −0.057, *t*-value = −1.398, df = 39.09, *p*-value = 0.17), although it was negative, indicating a slight decrease from pre- to post-intervention scores (after accounting for the repeated measurements for each educator). The educators’ scores remained consistent on the pre- and post-intervention surveys, there was less variability in responses for a particular educator (SD = 0.18) compared to between educators (SD = 0.32).

Lastly, for compassion fatigue (also sometimes referred to as secondary traumatic stress), there was a significant decrease in the scores of the post-intervention survey compared to the pre-intervention survey, after accounting for the repeated measurements for educators ($\overset{ˆ}{\beta }$ = −0.289, *t*-value = −4.291, df = 39.29, *p*-value = 0.0001). This decrease in scores did not depend on the educators’ age, gender, number of sessions attended, or ACE score.

#### 3.1.13. Educator Sense of Self-Efficacy (Short Form): TSSE

For the educators’ sense of self-efficacy survey instrument, there were also three subscales, which were analyzed separately. These subscales included feelings of self-efficacy in student engagement, instructional strategies, and classroom management. For all three subscales, the intervention did not seem to impact the educators’ sense of self-efficacy, but participants’ ACE scores did impact the scores, on average, for student engagement and classroom management.

For student engagement, on average, there was no difference between scores from the pre- to post-intervention survey, after accounting for the educator’s ACE score and the repeated measurements (*t*-value = −1.258, df = 38.73, *p*-value = 0.2159). However, as ACE scores increased, the educators’ sense of self-efficacy in student engagement scores also increased (*t*-value = 2.184, df = 41.07, *p*-value = 0.0348). This indicates that the higher the self-reported adverse childhood experience score of an educator, the more likely they are to feel a sense of self-efficacy in student engagement, regardless of age, gender, and the number of sessions attended.

On the “efficacy in instructional strategies” subscale, none of the variables measured seemed to explain the average score for these items; in particular, the timing variable was not important to the model (*t*-value = −0.033, df = 40.34, *p*-value = 0.974), although it was negative, indicating a very slight decrease from pre- to post-intervention scores (after accounting for the repeated measurements for each educator). The educators’ scores remained consistent on the pre- and post-intervention surveys, and there was less variability in responses for a particular educator (SD = 0.63) compared to between educators (SD = 0.85). Thus, because many educators rated their scores near “quite a bit” on the Likert scale of this instrument, the intervention did not impact the educators’ sense of self-efficacy in instructional strategies, regardless of their age, gender, or the number of intervention sessions they attended.

Finally, for classroom management, there was also no difference between scores from the pre- to post-intervention surveys, after accounting for the educators’ ACE score and the repeated measurements (*t*-value = −1.059, df = 39.98, *p*-value = 0.296). However, just as other measures have shown, as participants’ ACE scores increased, on average, the educators’ sense of self-efficacy in classroom management score also increased (*t*-value = 2.085, df = 43.05, *p*-value = 0.043). This illustrates that, as an educator’s ACE score increases, they are more likely to feel a sense of self-efficacy in classroom management, regardless of age, gender, or the number of intervention sessions they attended.

Tables 3 and 4 include the results from the final models for all scales below, indicating significant interactions found between pre- and post-intervention measures.

### 3.2. Psychophysiological Outcomes: Heart Rate Variability (HRV)

#### 3.2.1. Preliminary Examination of the Data

Preliminary analysis of student data (*n* = 39) indicated that 4 students were missing data at pre-intervention and 7 students were missing data at post-intervention resulting in a final sample of *n* = 32 students with complete pre- and post-intervention data. Moreover, there was one student whose RMSSD value at pre-intervention was greater than 3.29 standard deviations above the mean. We excluded this data point from further analysis and used pairwise analysis for all paired-sample *t*-tests. See Table 5 for means for HR, HF-HRV, and RMSSD across pre- and post-intervention sessions.

Preliminary analysis of educator data (*n* = 34) indicated that 1 educator was missing data at pre-intervention and 5 educators were missing data at post-intervention, resulting in a final sample of *n* = 29 educators with complete pre- and post-intervention data. Moreover, there was one educator whose RMSSD value at post-intervention was greater than 3.29 standard deviations above the mean. We excluded this data point from further analysis and used pairwise analysis for all paired-sample *t*-tests.

#### 3.2.2. Student Pre- to Post-Intervention Analysis

We conducted a one-tailed, paired-sample *t*-test for each student’s dependent variables (mean HR, HF-HRV, and RMSSD) with intervention time points (pre- and post-intervention) as independent variables. The results showed no significant change in mean HR (*t*-value = −0.82, df = 27, *p*-value = 0.21), HF-HRV (*t*-value = 0.91, df = 27, *p*-value = 0.19), and RMSSD (*t*-value = 1.23, df = 26, *p*-value = 0.12) from pre- to post-intervention.

#### 3.2.3. Educator Pre- to Post-Intervention Analysis

Just as with students, again, we conducted a one-tailed, paired-sample *t*-test for each educator’s dependent variables (mean HR, HF-HRV, and RMSSD) with intervention time points (pre- and post-intervention) as independent variables. The results showed no significant change in the mean HR (*t*-value = −1.45, df = 27, *p*-value = 0.08), HF-HRV (*t*-value = −0.45, df = 27, *p*-value = 0.33), and RMSSD (*t*-value = 1.01, df = 26, *p*-value = 0.16) from pre- to post-intervention.

#### 3.2.4. Psychophysiological Outcomes: Cortisol Levels

Cortisol levels were compared across all three experimental groups; broadly, the delivery modality seemed to play a critical role in improving cortisol levels. The only group that had a significant reduction in cortisol post-intervention was the student group (in the larger school district) who received the intervention in person. When controlling for baseline cortisol levels, this group (group L, to indicate the larger district), had a 0.19 μg/dL reduction in cortisol, on average, at post-intervention (*p*-value = 0.03). No pre–post differences in cortisol levels were observed in either of the virtual yoga intervention groups (students or adults). However, educators (group T) who received the virtual yoga intervention had significantly lower cortisol levels post-intervention compared to students who received the virtual yoga intervention (group S, to indicate the smaller school district) when controlling for baseline cortisol levels.

**Figure 2.**
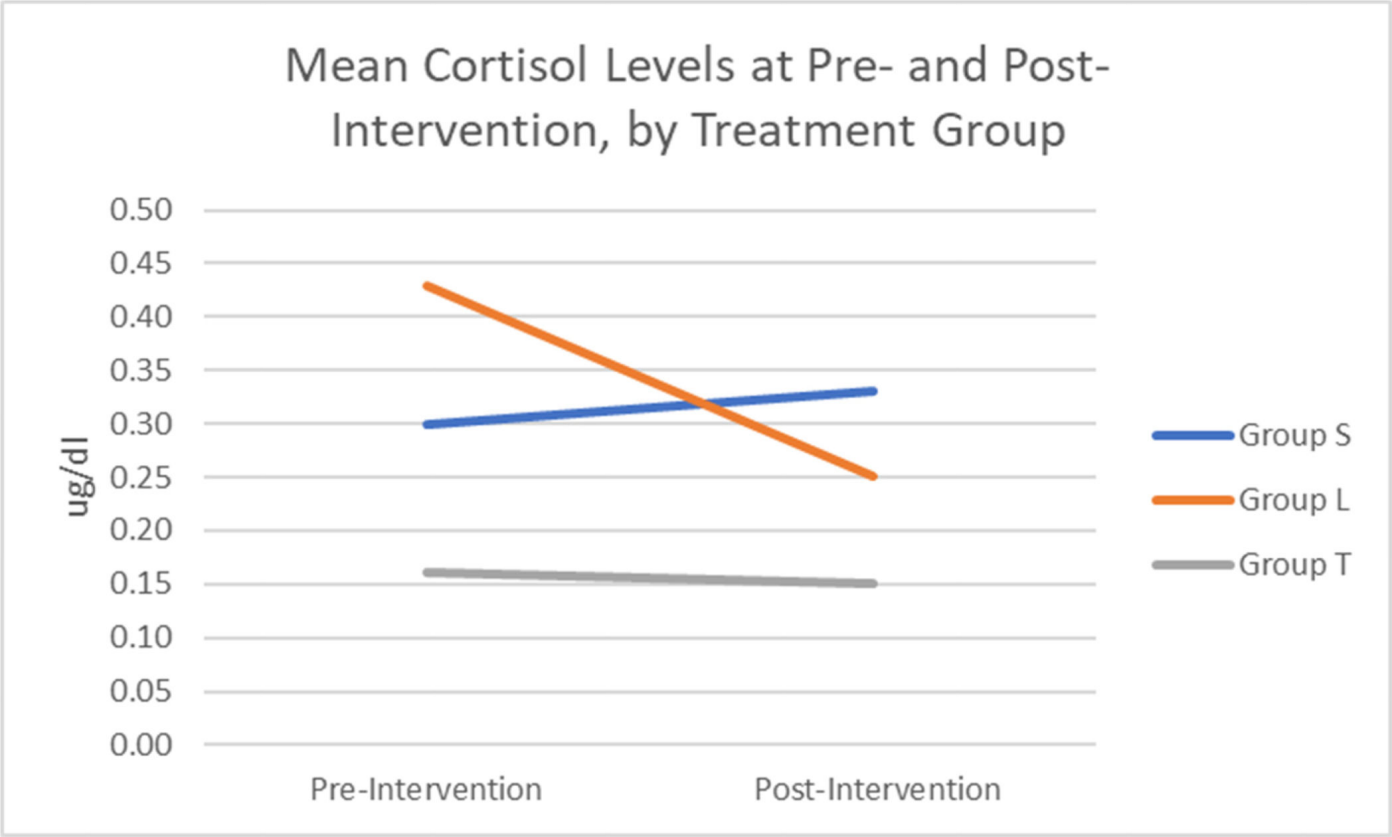
Mean cortisol levels by treatment group.

No dose–response association was observed between the number of yoga sessions attended and post-intervention cortisol levels in any experimental group, and no differential effect was observed when comparing pre- and post-cortisol levels by gender in any group. Figure 2 illustrates the changes in cortisol levels across all three groups, and Table 6 displays mean scores and *p*-values of cortisol levels.

**Figure 3.**
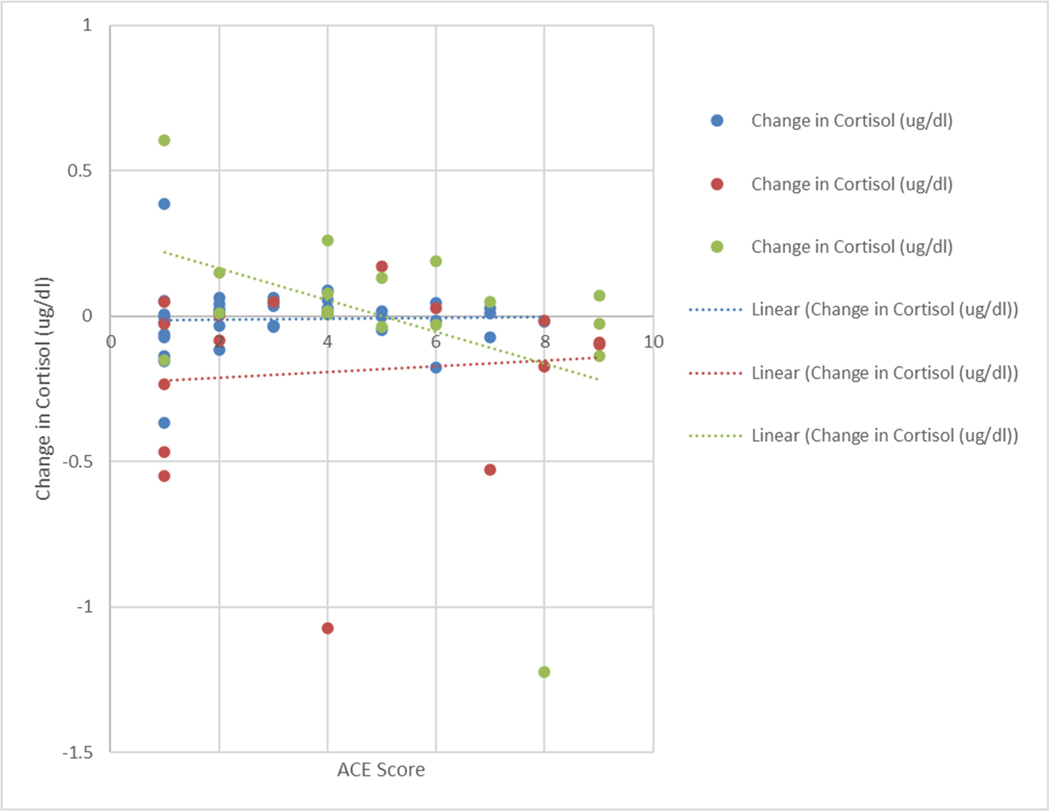
Association between adverse childhood event (ACE) scores and cortisol levels, post-intervention.

Associations between participant adverse childhood experience (ACE) scores and cortisol levels pre/post-intervention were also examined. Interestingly, in the geographically isolated group of students (group G), there was a borderline significant association between ACE scores and changes in cortisol levels post-intervention. The higher the student’s ACE score, the more their cortisol level decreased after the intervention; for every 1-unit increase in the ACE score among students in group G, there was a −0.02 ug/dL decrease in cortisol at post-intervention, on average, when controlling for baseline cortisol levels and gender (*p*-value = 0.08). No associations between ACE scores and change in cortisol were observed amongst the other intervention groups (the larger school district group of students or the educators). Figure 3 below illustrates associations between ACE scores and changes in cortisol levels across each experimental group.

Additionally, as part of the data collected at each cortisol collection, study participants were asked questions to self-report sleep quality and duration at the time of collection. While there were no statistically significant differences in pre- vs. post-intervention sleep duration, self-reported sleep quality at post-intervention appears to have improved in the in-person student yoga intervention group (group L). Again, educators who self-reported illness at post-intervention may have impacted their self-reported sleep quality and duration at the final cortisol collection. Table 7 below shows percentages of self-reported sleep quality pre-intervention as compared to post-intervention.

#### 3.2.5. Student and Educator Qualitative Feedback

Broadly, students shared mixed reactions (positive and negative) to their yoga classes, regardless of the delivery modality. Some positive sample responses included the following:

- “It lowered my stress a lot and made me feel very relaxed”.

- “It was good to be able to focus on myself and how I was feeling at the time, sometimes life is so busy you do not get a chance to process things and so it gave me time to process”.

Sample negative responses included the following:

- Student looked forward to “NOT doing yoga in the future” (*emphasis added by student*).

- Others noted that the yoga classes made them feel “sore” and “stressed”.

These mixed reactions mirror those of students in previous iterations of this study [38–43]. This intervention tends to polarize students in that they either enthusiastically support the intervention, or they clearly do not enjoy the sessions. Therefore, just as choice is critical in meeting student learning needs, the implications for school-based student mental health interventions seem to highlight the importance of choice. Research indicates that while some mental health interventions are incredibly helpful to some, they are not beneficial for others [8], so our conclusions from this study mirror these findings: while the results from this study are very positive, participant preference and enjoyment must be considered in school-based mental health programming for adolescents [37–42].

Unlike both student groups, educator responses to the open-response surveys given were overwhelmingly positive. Sample responses included the following:

- “I am appreciative that someone is attempting to do anything to make life better for an educator”.

- “I think doing yoga definitely helps me to be more patient at school especially when hard situations arise. I notice that I also feel more awake and ready for the day after doing yoga in the morning”.

- “‶ my general calm mood in response to students’ chaos helped me feel calmer and more relaxed. I also found myself teaching my students a lot of the moves that I learned that morning as a brain break”.

The very positive feedback provided by educator participants echoes that of previous research iterations [34], which indicates that this intervention may be more readily accepted by adults than student participants.

## 4. Discussion

The current study makes an incremental contribution to understanding the effectiveness of implementing trauma-informed yoga interventions in rural communities and schools in several ways. First, the results show mixed support for the potential impact the intervention may have on physical (cortisol and heart rate variability) and mental health (depression, anxiety, resilience, and sleep) for youth and educators. Second, intervention effectiveness may depend on whether it is provided remotely versus in-person. Third, findings suggested that the interactive nature of adverse childhood experiences, gender, and intervention participation may play a significant role in mental health outcomes improvement following the intervention. Finally, the intervention may have different benefits (or lack thereof) for youth and educators that will need to be further explored in future research. We delineate the findings in the context of past theory and research and their implications for practice separately for youth and educators in the next sections.

### 4.1. Student Discussion and Implications

In examining the mental health outcomes for students, multiple interactions were found, including students’ ACE scores, gender, time, and the number of sessions attended (depending on the measure). There were multiple statistically significant findings across both groups, as outlined above. These findings suggest that there were multiple potential factors that influenced how the students responded to the intervention. In particular, students with higher ACE scores responded differently to the intervention than those with lower ACE scores. For some survey measures, in addition to ACE scores, male and female students or students from different districts had different changes in the mean survey measure. So, while it is typically the case that the intervention was most impactful for rural, female students with high ACE scores, this was not consistent across all outcomes (anxiety, depression, and sleep). While differences were found among the student population for resilience, no difference was found due to the intervention after accounting for the characteristics of the students involved in the intervention.

In terms of physical health outcomes, students in the less rural school district, who received the in-person yoga sessions, had much lower cortisol levels than students who received the remotely delivered yoga. However, students in the more remote school district who had higher ACE scores did have lower cortisol at post-intervention. Additionally, students in the remote district, who received the remotely delivered yoga, had better mental health outcomes reported on surveys than students who received in-person yoga. Neither group (in-person or remotely delivered yoga) showed any significant changes in their heart rate variability measures. Self-reported sleep quality also improved across both districts, but the larger district (in-person delivery) reported far greater improvements in sleep quality. This may suggest that an in-person yoga intervention has more physical health benefits in terms of stress levels than a remotely delivered intervention, but perhaps a remotely delivered intervention may have a greater impact on mental health. However, concrete conclusions cannot be drawn from these data, as we did not randomly assign students to an intervention modality; further, in-person delivery may encourage students to engage more fully in the sessions than a remotely delivered intervention.

These findings mirror those of previous iterations of our study [37–42], which also had similar outcomes; a dose–response relationship appears to be indicated in terms of the number of sessions being correlated to a greater improvement in mental health outcomes. Additionally, females tended to have better outcomes than males, and students with higher ACE scores received better mental health benefits from the intervention than those with lower ACE scores [37–42]. However, it is still worth noting that this intervention did benefit students regardless of whether they had a low or high ACE score. Finally, student resilience outcomes continued to be mixed in this study, similar to our previous studies; in general, improvements in resilience were reported, but males with higher ACE scores exhibited decreased resilience. One potential explanation for this difference is that yoga increases interoception and self-reflection [65]; therefore, it is possible that students may become more self-aware of their own emotions and needs throughout this intervention, thus having an impact on perceived resilience.

Overall, there were mixed findings in our student groups, depending on whether the outcome was mental or physical health, as well as how the intervention was delivered. It is clear that there were benefits for both intervention groups, but further research is needed to explore why there was a difference between cortisol outcomes and mental health measures between the two groups. What stands out in this study, as well as in our previous studies, is the continued correlation between improved outcomes for students with high ACE scores as compared with students with lower ACE scores; this indicates that this trauma-informed intervention holds promise for mitigating childhood trauma among rural adolescents. Our research also supports the existing literature indicating mental and physical health outcomes using a yoga intervention for youth [8,24–26].

### 4.2. Educator Discussion and Implications

The findings showed there were no intervention effects on physical health outcomes for educators in terms of cortisol levels or heart rate variability. It is worth noting, however, that many of the educators self-reported illness at the post-intervention cortisol collection, so this may have impacted their cortisol levels. In comparison with our cortisol findings for students, some research indicates that a lack of improved cortisol may be a normal finding for adults [65–70], as noted by Galvan who asserted that “previous work has shown that, under identical stress conditions, teens show greater cortisol release than adults” [71]. Regarding heart rate variability, one potential explanation for this finding is that resting heart rate variability may be more entrained and difficult to change in such a short period of time. Past studies that showed significant effects were given at longer in-session durations (e.g., 60–90 min), greater frequency (5 days a week), and for longer intervention durations (10 weeks to 6 months); that is, it may take more practice or consistency in practice to change biologically based parasympathetic functioning [72–74]. Future research should explore trauma-informed interventions for longer durations and at greater frequency.

Similar to the mental health outcome findings with students, intervention effects may depend on the educators’ ACEs, gender, time, and the number of sessions attended. As outlined above, the results demonstrated mental health improvements across all genders. Further, just as with students, females showed greater improvements than males, and there appeared to be a correlation between greater improvements in mental health outcomes and higher participant ACE scores, again highlighting the potential of this intervention for mitigating childhood trauma, regardless of the age of the participant.

However, the effect of the intervention on career satisfaction seemed to differ depending on the domain, which also echoes our previous study with educators [42]. While burnout levels and compassion satisfaction generally improved, compassion fatigue scores declined; teachers also reported varying levels of self-efficacy in their classrooms post-intervention when compared with pre-intervention scores. Again, similar to student findings, participant ACEs seem to have an impact on these outcomes. These findings should be interpreted with caution, however, as there are many external variables that can impact a teacher’s career satisfaction on a daily basis; examples may include but are not limited to challenges with student behaviors, educator illness (which was self-reported by many teachers during the post-survey cortisol collection), or pressure from administration [75,76]. While additional study is needed to determine why there were mixed outcomes of career satisfaction measures amongst teachers, it is abundantly clear through this study and our previous study that educator wellness initiatives are critical for supporting educator well-being and career burnout levels [75,76].

### 4.3. Study Limitations

While there were many positive outcomes discovered with this intervention, it is important to note the limitations of this study. Given our very rural context, our sample was quite limited; additionally, while ethnic data were not formally collected as part of this experiment, our sample was quite homogenous. Thus, these findings cannot indicate generalizability to a larger and/or more diverse sample. Further, as stated earlier, a blizzard impeded the team’s ability to collect the heart rate variability data at the most remote school location, so these data were missing from our adult sample. Finally, at the time of our baseline data collection for the geographically isolated school, we discovered that a group of our student participants were also concurrently enrolled in another mental health intervention. Therefore, it is difficult to ascertain whether any mental health improvements in these students resulted from our intervention, the other intervention, or a combination of the two programs.

### 4.4. Recommendations for Future Research

While this study and the arc of the findings from previous iterations of this study [37–42] are very exciting and indicate tremendous potential for its use to benefit the mental and physical health of both students and educators, our population was very limited in terms of both diversity and sample size. The demographics of rural Montana, along with the nature of research in remote settings, provided unique challenges in both data collection as well as a more representative participant group. Therefore, the logical progression of this research would be to hold this study in a larger school district with an appropriate sample size for increased statistical power. However, one must not overlook the distinct needs and the contextual importance of working with rural schools in America, so findings from a more urban setting (for example) should be translated to rural contexts with an abundance of caution.

While the physical and mental health outcomes across all of our participant groups were mixed, the general mental health improvements across all groups, particularly amongst females and participants with the highest ACE scores, continue to contribute to the growing body of research for the promise of somatic interventions for mitigating trauma [19,77]. As a burgeoning area of research, trauma-informed yoga continues to exhibit its potential in improving the lives of traumatized individuals within many different age ranges, and the positive outcomes in a remotely delivered intervention have exciting implications for scalability and accessibility for those living in the most rural areas (who would not have access to such mental health interventions otherwise) [20].

## 5. Conclusions

The COVID-19 pandemic was a collective trauma experienced by everyone, and its impacts have been long-lasting; therefore, mental health and well-being must be addressed as students and educators adapt to our “new normal”. As the importance of trauma-informed, school-based interventions continues to gain momentum, it is important to research and explore options that provide benefits in the educational setting. Specifically, in rural communities, it is vitally important to continue exploring innovative, promising practices to foster student success but, more importantly, to bolster their mental and physical well-being.

## Supplementary Material

Supplementary Material

## Figures and Tables

**Table 1.** Initial paired *t*-test results for student surveys.

	Post-Intervention				Pre-Intervention			

	S		L		S		L	

	Mean	SD	Mean	SD	Mean	SD	Mean	SD
CD-RISC	25.56	7.76	27.33	6.06	26.64	8.17	25.68	6.52
GAD-7	8.22	4.57	6.06	5.02	9.45	5.62	7.84	5.36
PHQ-9	8.67 *	5.86	4.92 *	4.69	11.84 **	7.02	6.47 **	4.60
PROMIS	20.32	12.07	17.63	9.05	25.73	9.05	23.11	8.14

Note:

* *p* < 0.05,

** *p* < 0.01; paired *t*-test results assuming equal variances between groups for the difference in means test between districts within an intervention time period, i.e., comparing only pre-intervention scores between districts.

**Table 2.** Final linear mixed model results for student surveys.

	CDRISC	GAD-7	PHQ-9	PROMIS
Intercept	3.386 *** (0.268)	0.335 + (0.188)	−0.076 (0.321)	−3.180 (2.600)
ACE Score (Baseline)	−0.145 * (0.060)	0.152 *** (0.036)	0.253 *** (0.065)	0.253 ** (0.077)
Time (Pre/Post)	0.041 (0.071)	0.159 (0.218)	−0.306 ** (0.103)	−0.433 ** (0.136)
Gender (Female)	−1.226 ** (0.391)	0.475 ** (0.165)	1.370 ** (0.440)	
Baseline ACE Score * Gender (Female)	0.189 * (0.077)		−0.207 * (0.082)	
Baseline ACE score * Time (Pre/Post)		−0.086 * (0.041)		
School District (Larger)			0.596 (0.394)	0.793 (0.540)
Baseline ACE Score * School (Larger)*			−0.229 * (0.088)	−0.243 * (0.101)
School (Larger) * Gender (Female)			−1.241 * (0.562)	
Baseline ACE Score * School (Larger) *				
Gender (Female)			0.251 * (0.110)	
Student Age				0.330 * (0.157)

Note:

+ *p* < 0.1,

* *p* < 0.05,

** *p* < 0.01,

*** *p* < 0.001; only terms found in at least one of the final models for the CD-RISC, GAD-7, PHQ-9, and PROMIS SD instruments are included in the table. Values are coefficient estimates with standard errors in parentheses.

**Table 3.** Final linear mixed model results for the educator mental health surveys.

	CDRISC	GAD7	PCL5	PHQ9	PROMIS
Intercept	3.119 *** (0.186)	1.130 *** (0.105)	0.977 *** (0.088)	0.928 *** (0.243)	3.355 *** (0.393)
ACE Score (Baseline)	0.049 (0.034)				0.123 * (0.055)
Time (Pre/Post)	0.327 ** (0.109)	−0.353 *** (0.102)	−0.367 *** (0.084)	0.234 (0.241)	−0.469 *** (0.125)
Gender (Female)	−0.461 * (0.185)			0.415 * (0.201)	
ACE Score × Post-Intervention	−0.082 ** (0.029)				
Number of Sessions Attended				−0.058 ** (0.022)	−0.080 * (0.038)
Gender (Female) × Post-Intervention				−0.559 * (0.252)	

Note:

* *p* < 0.05,

** *p* < 0.01,

*** *p* < 0.001; only terms found in at least one of the final models for the CD-RISC, GAD-7, PCL-5, PHQ-9, and PROMIS SD instruments are included in the table. Values are coefficient estimates with standard errors in parentheses.

**Table 4.** Final linear mixed model results for educator career satisfaction surveys.

	ProQOL CS	ProQOL B	ProQOL STS	SES SE	SES IS	SES CM
Intercept	2.782 ***	3.553 ***	2.484 ***	6.015 ***	7.119 ***	6.631 ***
	(0.293)	(0.101)	(0.078)	(0.291)	(0.159)	(0.295)
ACE Score (Baseline)	0.111 **	−0.017		0.164 *		0.162 *
	(0.041)	(0.027)		(0.075)		(0.078)
Time (Pre/Post)	−0.101	−0.057	−0.289 ***	−0.218	−0.005	−0.140
	(0.089)	(0.041)	(0.067)	(0.173)	(0.142)	(0.132)

Note:

* *p* < 0.05,

** *p* < 0.01,

*** *p* < 0.001; only terms found in at least one of the final models for the ProQOL and educators’ sense of self-efficacy instruments are included in the table. Coefficients are on the first line; standard errors are in parentheses.

**Table 5.** Mean heart rate, HF-HRV, and RMSSD across pre- and post-intervention sessions.

	Pre-Intervention	Post-Intervention
Student Data		
Mean Heart Rate	71.65 (12.06)	71.17 (10.46)
HF-HRV	6.74 (1.09)	6.85 (0.97)
RMSSD	63.86 (39.87)	64.82 (32.42)

Educator Data		
Mean Heart Rate	68.46 (10.99)	70.87 (12.41)
HF-HRV	6.05 (1.37)	6.00 (1.37)
RMSSD	40.88 (25.86)	43.04 (45.78)

Note: HF-HRV = high frequency—heart rate variability; RMSSD = root mean squared of successive differences. Missing data for the student sample resulted in *n* = 35 for pre-intervention and *n* = 32 for post-intervention; missing data for the educator sample resulted in *n* = 33 for pre-intervention and *n* = 29 for post-intervention. Mean values are followed by SD in parentheses.

**Table 6.** Mean cortisol level pre- to post-intervention differences by treatment group.

Group Intervention	Pre-Intervention	Post-Intervention	Mean Difference	*p*-Value
Small School	0.30 (0.40) *n* = 22	0.33 (0.20) *n* = 17	0.00 (0.36) *n* = 17	0.98
**Large School**	**0.43 (0.38)** ***n* = 17**	**0.25 (0.13)** ***n* = 17**	**−0.19 (0.32)** ***n* = 16**	**0.03**
Teachers	0.16 (0.11) *n* = 43	0.15 (0.11) *n* = 38	−0.01 (0.11) *n* = 37	0.56

Note: Bolded group is statistically significant at *p* < 0.05; *p*-value corresponds to paired *t*-tests.

**Table 7.** Self-reported sleep quality of participants, pre- vs. post-intervention.

Group	Self-Reported Sleep Quality	Pre-Intervention N (Percent)	Post-Intervention N (Percent)
Smaller School	Did not sleep well	17 (77.3%)	11 (68.8%)

	Slept well	5 (22.7%)	5 (31.3%)

Larger School	Did not sleep well	8 (47.1%)	2 (11.8%)

	Slept well	9 (52.9%)	15 (88.2%)

Teachers	Did not sleep well	27 (61.4%)	19 (48.7%)

	Slept well	17 (38.6%)	20 (51.3%)

## Data Availability

Given that some of the study participants were minors, study data is unavailable due to privacy restrictions.
