# Supplementary material for: From Surviving to Thriving: A Trauma-Informed Yoga Intervention for Adolescents and Educators in Rural Montana
Source: Educ Sci (Basel). Author manuscript; Available in PMC 2024 Dec 31. (PMC11687386; doi:10.3390/educsci14121394)
Supplement: Supplementary Material [file NIHMS2044203-supplement-Supplementary_Material.docx]

## Educator models

### Generalized Anxiety Disorder – 7 (GAD-7) Final Model

Linear mixed model fit by REML. t-tests use Satterthwaite's method [
lmerModLmerTest]
Formula: gad7 ~ time + (1 | teachID)
 Data: gad7_teachData_long_mean

REML criterion at convergence: 163.4

Scaled residuals:
 Min 1Q Median 3Q Max
-1.7207 -0.5297 -0.1226 0.4430 2.2064

Random effects:
 Groups Name Variance Std.Dev.
 teachID (Intercept) 0.2750 0.5244
 Residual 0.2078 0.4558
Number of obs: 83, groups: teachID, 44

Fixed effects:
 Estimate Std. Error df t value Pr(>|t|)
(Intercept) 1.1299 0.1047 60.6168 10.787 9.86e-16 ***
time -0.3529 0.1019 37.3749 -3.462 0.00136 **
---
Signif. codes: 0 '***' 0.001 '**' 0.01 '*' 0.05 '.' 0.1 ' ' 1

Correlation of Fixed Effects:
 (Intr)
time -0.442

Analysis of Deviance Table (Type II Wald chisquare tests)

Response: gad7
 Chisq Df Pr(>Chisq)
time 11.984 1 0.0005366 ***
---
Signif. codes: 0 '***' 0.001 '**' 0.01 '*' 0.05 '.' 0.1 ' ' 1


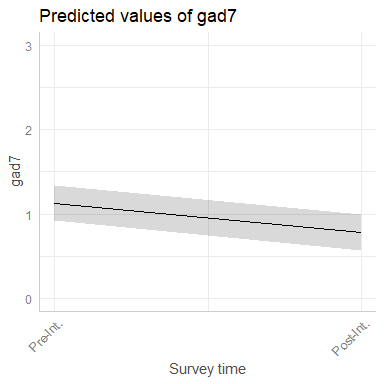


Figure S1: Predicted mean GAD-7 score based on the final model that includes a main effect for time, and accounts for the repeated measurements for teachers.

# Predicted values of gad7

time | Predicted | 95% CI
-----------------------------
 0 | 1.13 | 0.92, 1.34
 1 | 0.78 | 0.56, 0.99


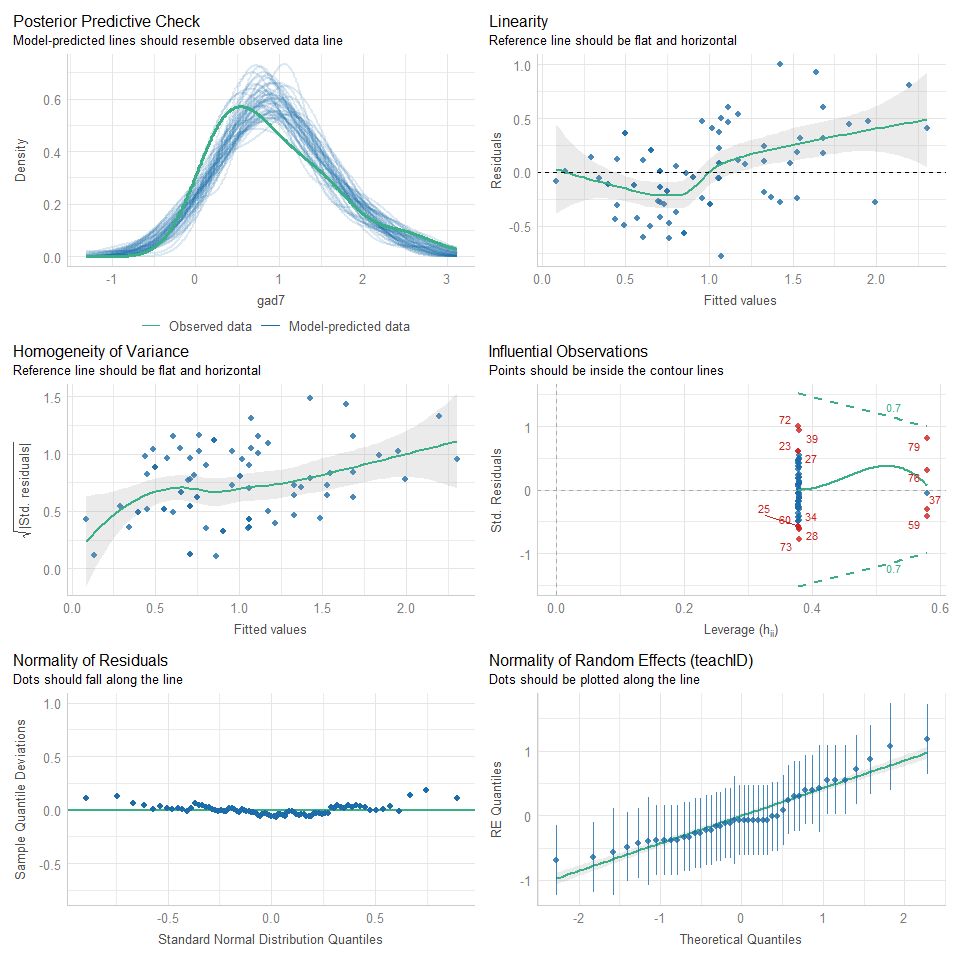


Figure S2: Model diagnostic plot for the GAD-7 response for the teachers’ data.

### Patient Health Questionnaire – 9 (PHQ-9) Final Model

Linear mixed model fit by REML. t-tests use Satterthwaite's method [
lmerModLmerTest]
Formula: phq9 ~ time * gen_cnf + numSess + (1 | teachID)
 Data: phq9_teachData_long_mean

REML criterion at convergence: 102.3

Scaled residuals:
 Min 1Q Median 3Q Max
-1.36882 -0.53951 0.00029 0.47055 2.54179

Random effects:
 Groups Name Variance Std.Dev.
 teachID (Intercept) 0.10554 0.3249
 Residual 0.09907 0.3148
Number of obs: 83, groups: teachID, 44

Fixed effects:
 Estimate Std. Error df t value Pr(>|t|)
(Intercept) 0.92817 0.24341 53.78424 3.813 0.000355 ***
time 0.23444 0.24104 44.80820 0.973 0.335961
gen_cnfFemale 0.41528 0.20102 60.91587 2.066 0.043099 *
numSess -0.05839 0.02162 44.23443 -2.700 0.009781 **
time:gen_cnfFemale -0.55911 0.25198 44.10164 -2.219 0.031690 *
---
Signif. codes: 0 '***' 0.001 '**' 0.01 '*' 0.05 '.' 0.1 ' ' 1

Correlation of Fixed Effects:
 (Intr) time gn_cnF numSss
time -0.263
gen_cnfFeml -0.599 0.345
numSess -0.651 -0.029 -0.151
tm:gn_cnfFm 0.262 -0.956 -0.379 0.011

Analysis of Deviance Table (Type II Wald chisquare tests)

Response: phq9
 Chisq Df Pr(>Chisq)
time 15.3718 1 8.829e-05 ***
gen_cnf 1.7516 1 0.185672
numSess 7.2913 1 0.006929 **
time:gen_cnf 4.9233 1 0.026497 *
---
Signif. codes: 0 '***' 0.001 '**' 0.01 '*' 0.05 '.' 0.1 ' ' 1


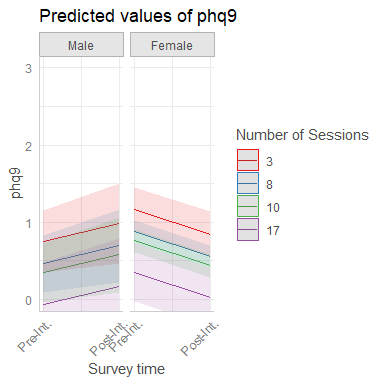


Figure S3: Predicted mean PHQ-9 score based on the final model that includes time, gender, and accounts for the repeated measurements for teachers.

# Predicted values of phq9

numSess: 3
gen_cnf: Male

time | Predicted | 95% CI
-----------------------------
 0 | 0.75 | 0.35, 1.16
 1 | 0.99 | 0.47, 1.50

numSess: 3
gen_cnf: Female

time | Predicted | 95% CI
-----------------------------
 0 | 1.17 | 0.89, 1.45
 1 | 0.84 | 0.55, 1.13

numSess: 8
gen_cnf: Male

time | Predicted | 95% CI
-----------------------------
 0 | 0.46 | 0.10, 0.82
 1 | 0.70 | 0.22, 1.17

numSess: 8
gen_cnf: Female

time | Predicted | 95% CI
-----------------------------
 0 | 0.88 | 0.73, 1.02
 1 | 0.55 | 0.40, 0.70

numSess: 10
gen_cnf: Male

time | Predicted | 95% CI
------------------------------
 0 | 0.34 | -0.03, 0.72
 1 | 0.58 | 0.09, 1.07

numSess: 10
gen_cnf: Female

time | Predicted | 95% CI
-----------------------------
 0 | 0.76 | 0.61, 0.91
 1 | 0.43 | 0.28, 0.59

numSess: 17
gen_cnf: Male

time | Predicted | 95% CI
------------------------------
 0 | -0.06 | -0.61, 0.48
 1 | 0.17 | -0.45, 0.79

numSess: 17
gen_cnf: Female

time | Predicted | 95% CI
------------------------------
 0 | 0.35 | -0.03, 0.73
 1 | 0.03 | -0.35, 0.40


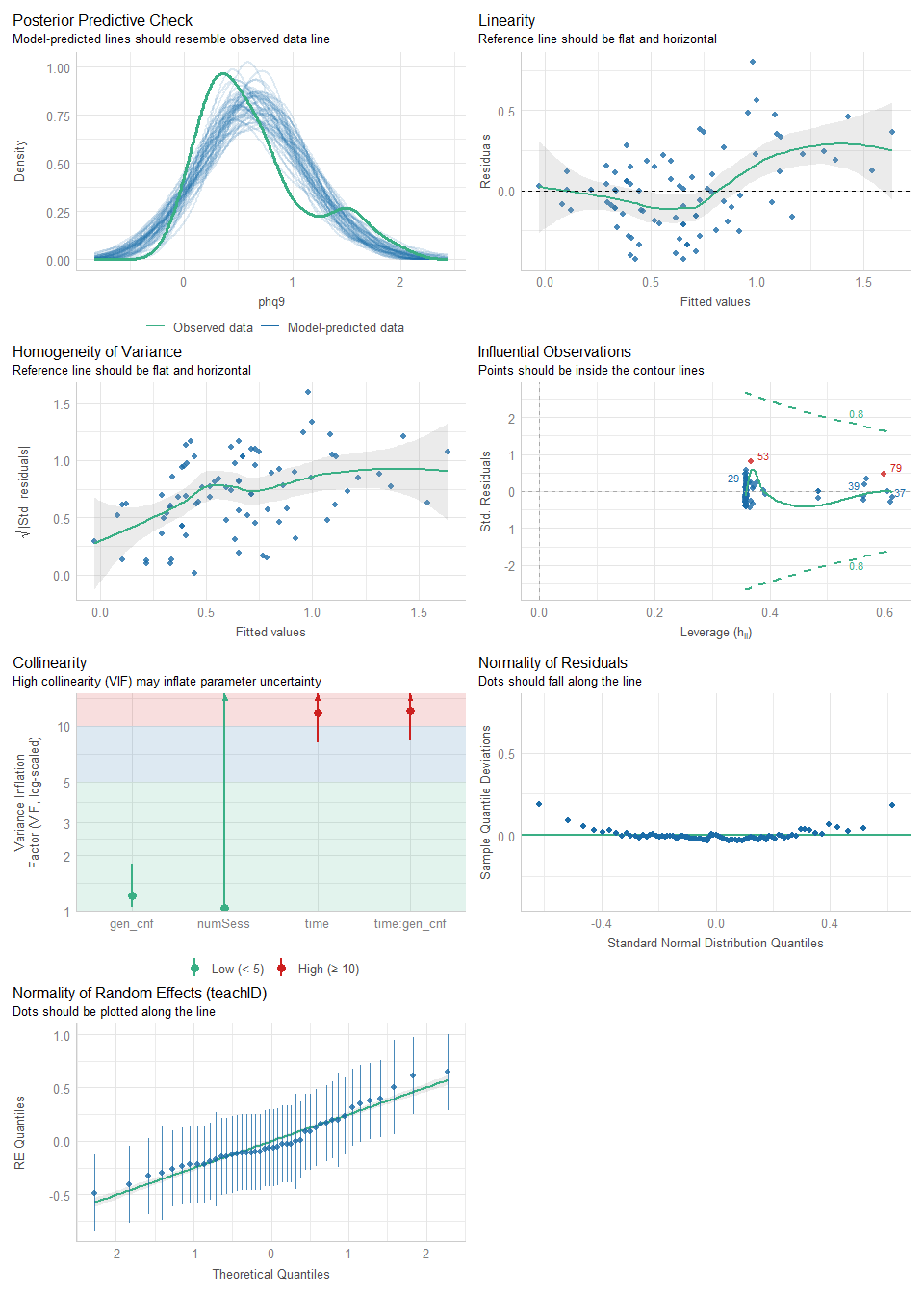


Figure S4: Model diagnostic plot for the PHQ-9 response for the teachers’ data.

### PTSD Checklist for DSM-5: Adult Version (PCL-5) Final Model

Linear mixed model fit by REML. t-tests use Satterthwaite's method [
lmerModLmerTest]
Formula: pcl5 ~ time + (1 | teachID)
 Data: pcl5_teachData_long_mean

REML criterion at convergence: 134.8

Scaled residuals:
 Min 1Q Median 3Q Max
-1.5051 -0.4814 -0.1211 0.3722 2.3387

Random effects:
 Groups Name Variance Std.Dev.
 teachID (Intercept) 0.2010 0.4483
 Residual 0.1425 0.3775
Number of obs: 83, groups: teachID, 44

Fixed effects:
 Estimate Std. Error df t value Pr(>|t|)
(Intercept) 0.97727 0.08836 60.84103 11.061 3.40e-16 ***
time -0.36712 0.08447 38.67546 -4.346 9.72e-05 ***
---
Signif. codes: 0 '***' 0.001 '**' 0.01 '*' 0.05 '.' 0.1 ' ' 1

Correlation of Fixed Effects:
 (Intr)
time -0.434

Analysis of Deviance Table (Type II Wald chisquare tests)

Response: pcl5
 Chisq Df Pr(>Chisq)
time 18.889 1 1.385e-05 ***
---
Signif. codes: 0 '***' 0.001 '**' 0.01 '*' 0.05 '.' 0.1 ' ' 1


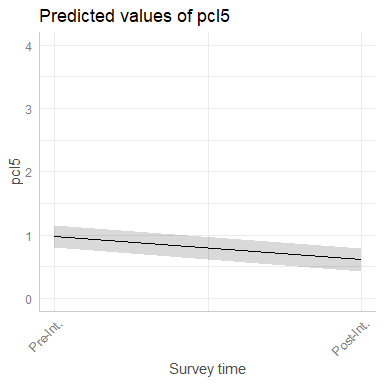


Figure S5: Predicted mean PCL-5 score based on the final model that includes a main effect for time, and accounts for the repeated measurements for teachers.

# Predicted values of pcl5

time | Predicted | 95% CI
-----------------------------
 0 | 0.98 | 0.80, 1.15
 1 | 0.61 | 0.43, 0.79


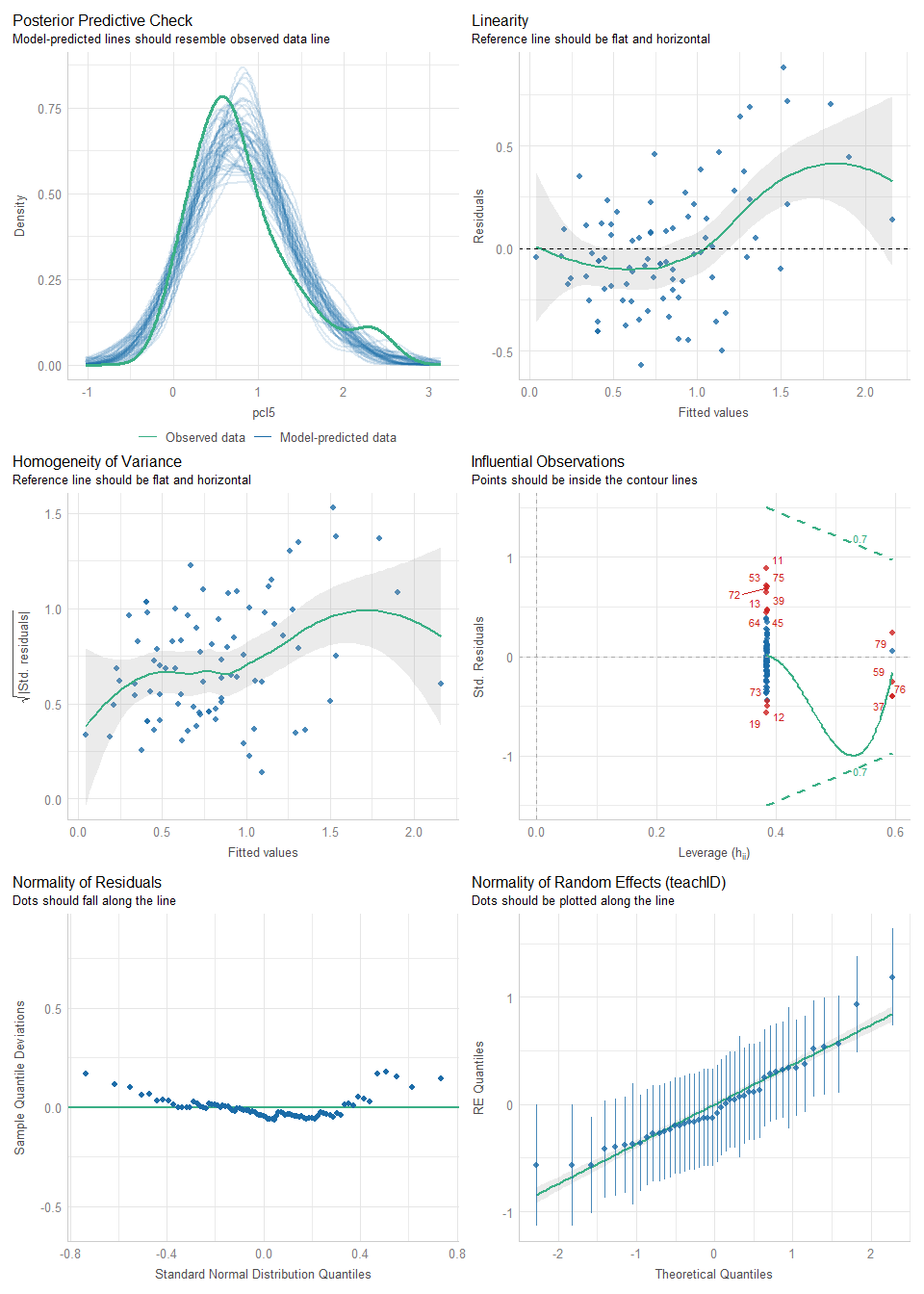


Figure S6: Model diagnostic plot for the PCL-5 response for the teachers’ data.

### Patient-Reported Outcomes Measurement Information System – Sleep Disturbance Short Form for Adults (PROMIS SD-SF-Adult) Final Model

Linear mixed model fit by REML. t-tests use Satterthwaite's method [
lmerModLmerTest]
Formula: promsd ~ ace1_1 + time + numSess + (1 | teachID)
 Data: promsd_teachData_long_mean

REML criterion at convergence: 198

Scaled residuals:
 Min 1Q Median 3Q Max
-2.62967 -0.52820 0.04953 0.52558 2.34056

Random effects:
 Groups Name Variance Std.Dev.
 teachID (Intercept) 0.3499 0.5915
 Residual 0.3113 0.5579
Number of obs: 83, groups: teachID, 44

Fixed effects:
 Estimate Std. Error df t value Pr(>|t|)
(Intercept) 3.35540 0.39294 46.18311 8.539 4.65e-11 ***
ace1_1 0.12276 0.05490 41.73738 2.236 0.030764 *
time -0.46921 0.12498 39.99083 -3.754 0.000553 ***
numSess -0.07966 0.03849 45.91146 -2.069 0.044163 *
---
Signif. codes: 0 '***' 0.001 '**' 0.01 '*' 0.05 '.' 0.1 ' ' 1

Correlation of Fixed Effects:
 (Intr) ace1_1 time
ace1_1 -0.449
time -0.080 -0.005
numSess -0.841 0.010 -0.073

Analysis of Deviance Table (Type II Wald chisquare tests)

Response: promsd
 Chisq Df Pr(>Chisq)
ace1_1 4.9993 1 0.0253583 *
time 14.0936 1 0.0001739 ***
numSess 4.2825 1 0.0385077 *
---
Signif. codes: 0 '***' 0.001 '**' 0.01 '*' 0.05 '.' 0.1 ' ' 1


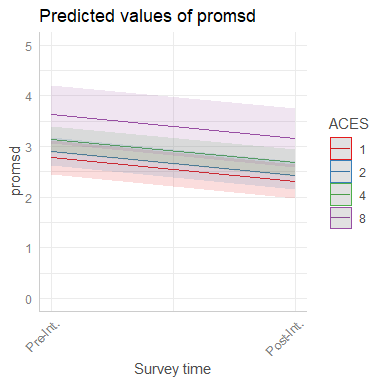


Figure S7: Predicted mean PROMIS score based on the final model that includes a main effect for time, ACES, number of sessions, and accounts for the repeated measurements for teachers.

# Predicted values of promsd

ace1_1: 1

time | Predicted | 95% CI
-----------------------------
 0 | 2.78 | 2.44, 3.11
 1 | 2.31 | 1.97, 2.65

ace1_1: 2

time | Predicted | 95% CI
-----------------------------
 0 | 2.90 | 2.63, 3.17
 1 | 2.43 | 2.15, 2.71

ace1_1: 4

time | Predicted | 95% CI
-----------------------------
 0 | 3.15 | 2.89, 3.40
 1 | 2.68 | 2.41, 2.94

ace1_1: 8

time | Predicted | 95% CI
-----------------------------
 0 | 3.64 | 3.06, 4.21
 1 | 3.17 | 2.59, 3.75

Adjusted for:
* numSess = 8.78


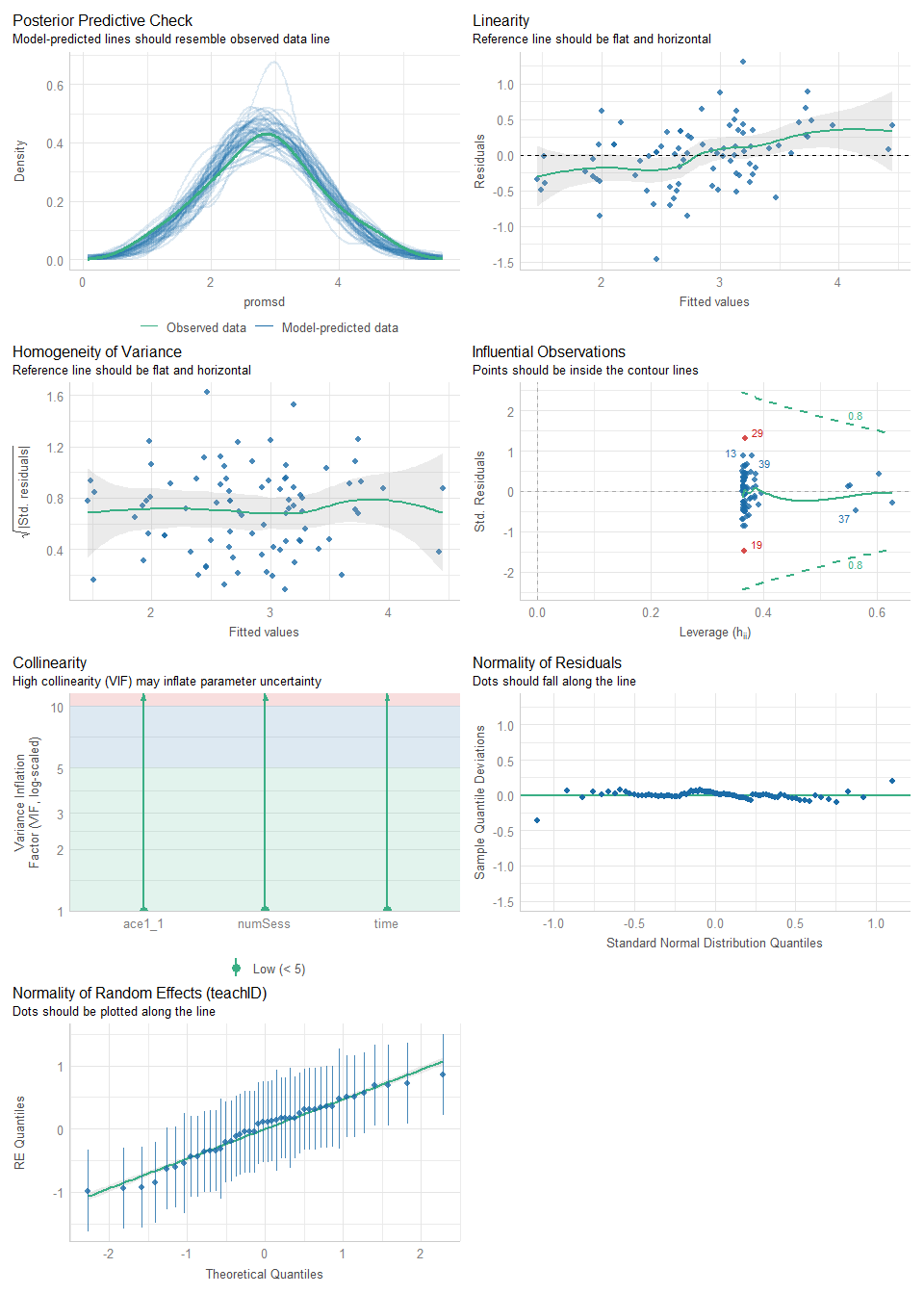


Figure S8: Model diagnostic plot for the PROMIS SD-SF response for the teachers’ data.

### Connor Davidson Resilience Scale (CD-RISC) Final model

Linear mixed model fit by REML. t-tests use Satterthwaite's method [
lmerModLmerTest]
Formula: cdrisc ~ ace1_1 * time + gen_cnf + (1 | teachID)
 Data: cdrisc_teachData_long_mean

REML criterion at convergence: 89.8

Scaled residuals:
 Min 1Q Median 3Q Max
-1.66385 -0.50019 -0.04184 0.52179 2.09853

Random effects:
 Groups Name Variance Std.Dev.
 teachID (Intercept) 0.12527 0.3539
 Residual 0.06439 0.2537
Number of obs: 83, groups: teachID, 44

Fixed effects:
 Estimate Std. Error df t value Pr(>|t|)
(Intercept) 3.11901 0.18650 49.83913 16.724 < 2e-16 ***
ace1_1 0.04948 0.03363 55.06327 1.471 0.14692
time 0.32719 0.10882 38.72969 3.007 0.00462 **
gen_cnfFemale -0.46089 0.18525 45.74476 -2.488 0.01655 *
ace1_1:time -0.08188 0.02915 38.89056 -2.809 0.00774 **
---
Signif. codes: 0 '***' 0.001 '**' 0.01 '*' 0.05 '.' 0.1 ' ' 1

Correlation of Fixed Effects:
 (Intr) ace1_1 time gn_cnF
ace1_1 -0.408
time -0.215 0.330
gen_cnfFeml -0.750 -0.189 -0.053
ace1_1:time 0.199 -0.382 -0.852 0.022


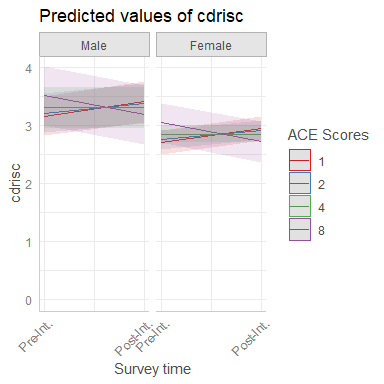


Figure S9: Predicted mean CD-RISC score based on the final model that includes a two-way interaction between time and ACES score, different intercepts for gender, and accounts for the repeated measurements for teachers. The predicted line for CD-RISC by time for an ACES score of 8 for males is based on a prediction assuming a linear relationship between ACES score and CD-RISC, although the maximum score for males that was observed was 4.

# Predicted values of cdrisc

ace1_1: 1
gen_cnf: Male

time | Predicted | 95% CI
-----------------------------
 0 | 3.17 | 2.82, 3.51
 1 | 3.41 | 3.06, 3.77

ace1_1: 1
gen_cnf: Female

time | Predicted | 95% CI
-----------------------------
 0 | 2.71 | 2.50, 2.91
 1 | 2.95 | 2.74, 3.16

ace1_1: 2
gen_cnf: Male

time | Predicted | 95% CI
-----------------------------
 0 | 3.22 | 2.88, 3.55
 1 | 3.38 | 3.04, 3.73

ace1_1: 2
gen_cnf: Female

time | Predicted | 95% CI
-----------------------------
 0 | 2.76 | 2.59, 2.92
 1 | 2.92 | 2.76, 3.09

ace1_1: 4
gen_cnf: Male

time | Predicted | 95% CI
-----------------------------
 0 | 3.32 | 2.96, 3.67
 1 | 3.32 | 2.96, 3.68

ace1_1: 4
gen_cnf: Female

time | Predicted | 95% CI
-----------------------------
 0 | 2.86 | 2.71, 3.00
 1 | 2.86 | 2.71, 3.00

ace1_1: 8
gen_cnf: Male

time | Predicted | 95% CI
-----------------------------
 0 | 3.51 | 3.01, 4.02
 1 | 3.19 | 2.67, 3.70

ace1_1: 8
gen_cnf: Female

time | Predicted | 95% CI
-----------------------------
 0 | 3.05 | 2.72, 3.39
 1 | 2.73 | 2.37, 3.08


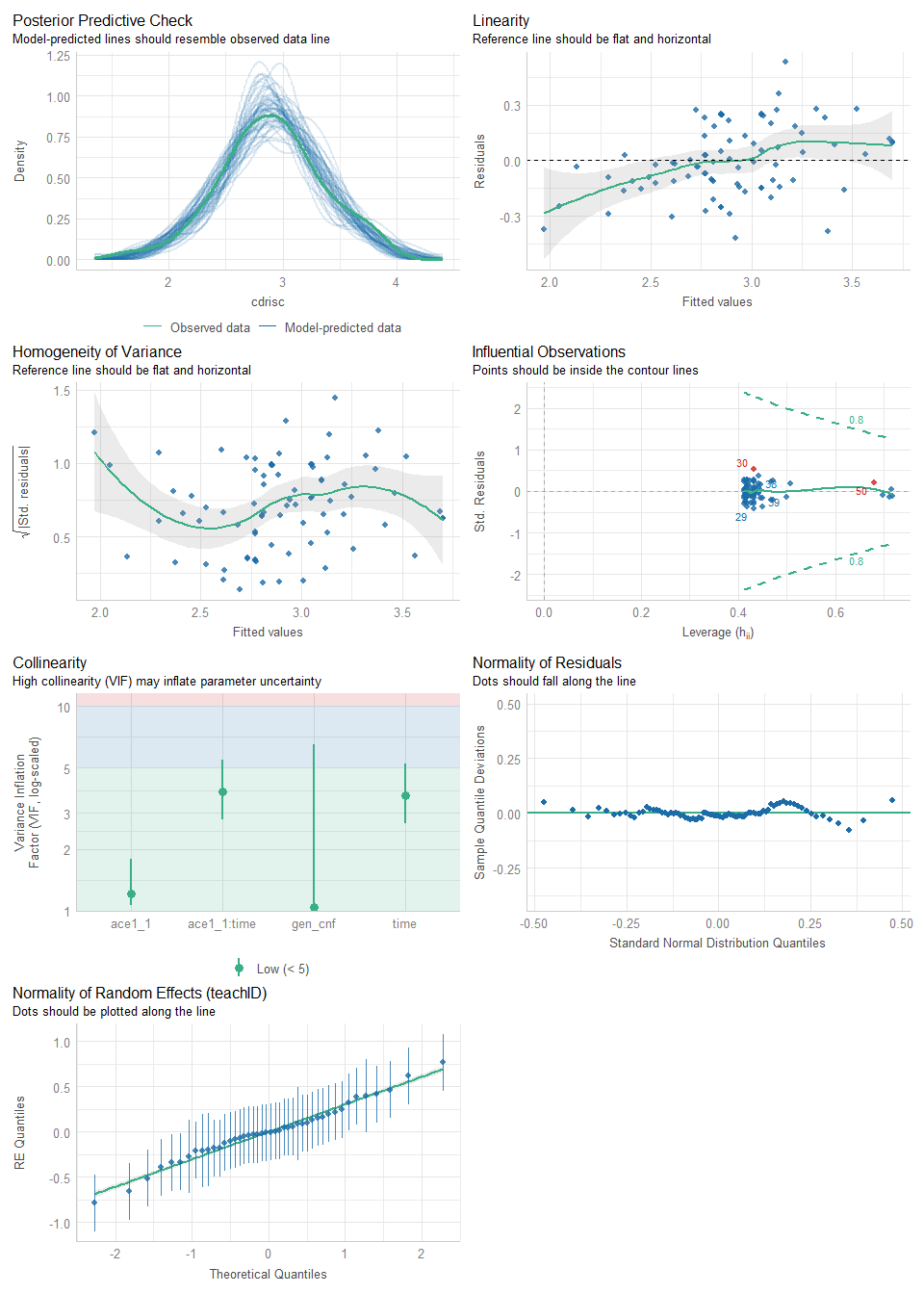


Figure S10: Model diagnostic plots for the CD-RISC response for the final model for the teachers’ data.

### Educator Career Satisfaction Professional Quality of Life Index (ProQOL)

#### Compassion Satisfaction Final Model

Linear mixed model fit by REML. t-tests use Satterthwaite's method [
lmerModLmerTest]
Formula: proqol_compSat ~ ace1_1 + time + numSess + (1 | teachID)
 Data: proqol_compSat_teachData_long_mean

REML criterion at convergence: 148.7

Scaled residuals:
 Min 1Q Median 3Q Max
-2.37633 -0.46368 -0.07856 0.50088 1.85366

Random effects:
 Groups Name Variance Std.Dev.
 teachID (Intercept) 0.2045 0.4522
 Residual 0.1589 0.3986
Number of obs: 83, groups: teachID, 44

Fixed effects:
 Estimate Std. Error df t value Pr(>|t|)
(Intercept) 2.78157 0.29343 42.90631 9.480 4.36e-12 ***
ace1_1 0.11051 0.04109 38.74766 2.689 0.01051 *
time -0.10065 0.08937 36.88046 -1.126 0.26733
numSess 0.07779 0.02875 42.69796 2.706 0.00975 **
---
Signif. codes: 0 '***' 0.001 '**' 0.01 '*' 0.05 '.' 0.1 ' ' 1

Correlation of Fixed Effects:
 (Intr) ace1_1 time
ace1_1 -0.451
time -0.076 -0.005
numSess -0.842 0.010 -0.071


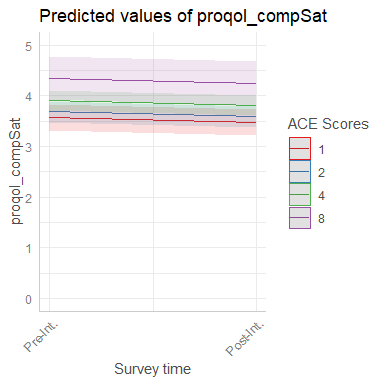


Figure S11: Predicted mean ProQOL Compassion Satisfaction score based on the final model that includes ACES score, time, number of sessions, and accounts for the repeated measurements for teachers. The predicted line for ProQOL Compassion Satisfaction by time for an ACES score of 8 for males is based on a prediction assuming a linear relationship between ACES score and ProQOL Compassion Satisfaction, although the maximum score for males that was observed was 4.

# Predicted values of proqol_compSat

ace1_1: 1

time | Predicted | 95% CI
-----------------------------
 0 | 3.58 | 3.33, 3.82
 1 | 3.47 | 3.22, 3.73

ace1_1: 2

time | Predicted | 95% CI
-----------------------------
 0 | 3.69 | 3.48, 3.89
 1 | 3.59 | 3.38, 3.79

ace1_1: 4

time | Predicted | 95% CI
-----------------------------
 0 | 3.91 | 3.72, 4.10
 1 | 3.81 | 3.61, 4.00

ace1_1: 8

time | Predicted | 95% CI
-----------------------------
 0 | 4.35 | 3.92, 4.78
 1 | 4.25 | 3.82, 4.68

Adjusted for:
* numSess = 8.78


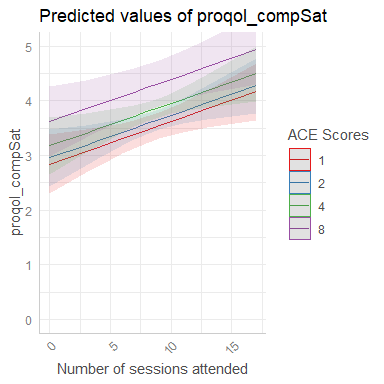


Figure S12: Predicted mean ProQOL Compassion Satisfaction score based on the final model that includes ACES score, time, number of sessions, and accounts for the repeated measurements for teachers. The predicted line for ProQOL Compassion Satisfaction by time for an ACES score of 8 for males is based on a prediction assuming a linear relationship between ACES score and ProQOL Compassion Satisfaction, although the maximum score for males that was observed was 4.

# Predicted values of proqol_compSat

ace1_1: 1

numSess | Predicted | 95% CI
--------------------------------
 0 | 2.84 | 2.30, 3.39
 4 | 3.16 | 2.80, 3.51
 7 | 3.39 | 3.13, 3.64
 9 | 3.54 | 3.31, 3.78
 17 | 4.17 | 3.64, 4.69

ace1_1: 2

numSess | Predicted | 95% CI
--------------------------------
 0 | 2.96 | 2.43, 3.48
 4 | 3.27 | 2.95, 3.59
 7 | 3.50 | 3.29, 3.71
 9 | 3.66 | 3.47, 3.84
 17 | 4.28 | 3.77, 4.78

ace1_1: 4

numSess | Predicted | 95% CI
--------------------------------
 0 | 3.18 | 2.66, 3.69
 4 | 3.49 | 3.17, 3.80
 7 | 3.72 | 3.52, 3.92
 9 | 3.88 | 3.70, 4.05
 17 | 4.50 | 4.00, 5.00

ace1_1: 8

numSess | Predicted | 95% CI
--------------------------------
 0 | 3.62 | 2.98, 4.26
 4 | 3.93 | 3.44, 4.42
 7 | 4.16 | 3.73, 4.59
 9 | 4.32 | 3.90, 4.74
 17 | 4.94 | 4.31, 5.58

Adjusted for:
* time = 0.47


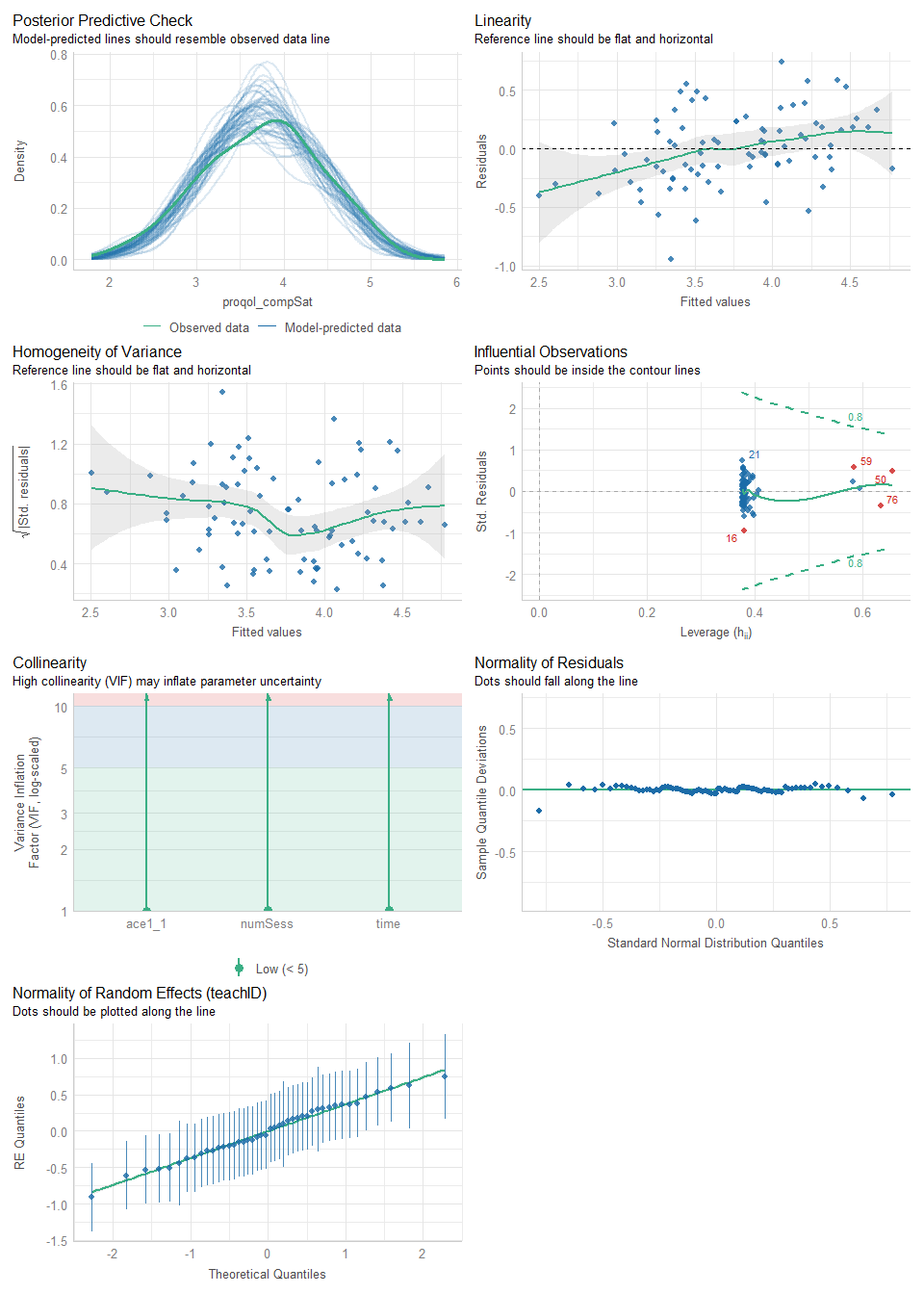


Figure S13: Model diagnostic plots for the ProQOL – Compassion satisfaction response for the final model for the teachers’ data.

#### Burnout Final Model

Linear mixed model fit by REML. t-tests use Satterthwaite's method [
lmerModLmerTest]
Formula: proqol_burn ~ time + (1 | teachID)
 Data: proqol_burn_teachData_long_mean

REML criterion at convergence: 42.5

Scaled residuals:
 Min 1Q Median 3Q Max
-2.17833 -0.61329 0.01683 0.48429 2.06028

Random effects:
 Groups Name Variance Std.Dev.
 teachID (Intercept) 0.1026 0.3203
 Residual 0.0325 0.1803
Number of obs: 83, groups: teachID, 44

Fixed effects:
 Estimate Std. Error df t value Pr(>|t|)
(Intercept) 3.49773 0.05541 53.26444 63.129 <2e-16 ***
time -0.05667 0.04054 39.08709 -1.398 0.17
---
Signif. codes: 0 '***' 0.001 '**' 0.01 '*' 0.05 '.' 0.1 ' ' 1

Correlation of Fixed Effects:
 (Intr)
time -0.329

Analysis of Deviance Table (Type II Wald chisquare tests)

Response: proqol_burn
 Chisq Df Pr(>Chisq)
time 1.954 1 0.1622


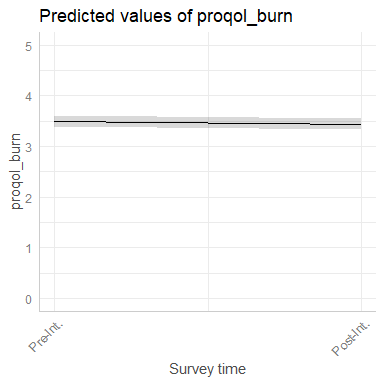


Figure S14: Predicted mean ProQOL Burnout score based on the final model that includes time and accounts for the repeated measurements for teachers.

# Predicted values of proqol_burn

time | Predicted | 95% CI
-----------------------------
 0 | 3.50 | 3.39, 3.61
 1 | 3.44 | 3.33, 3.55


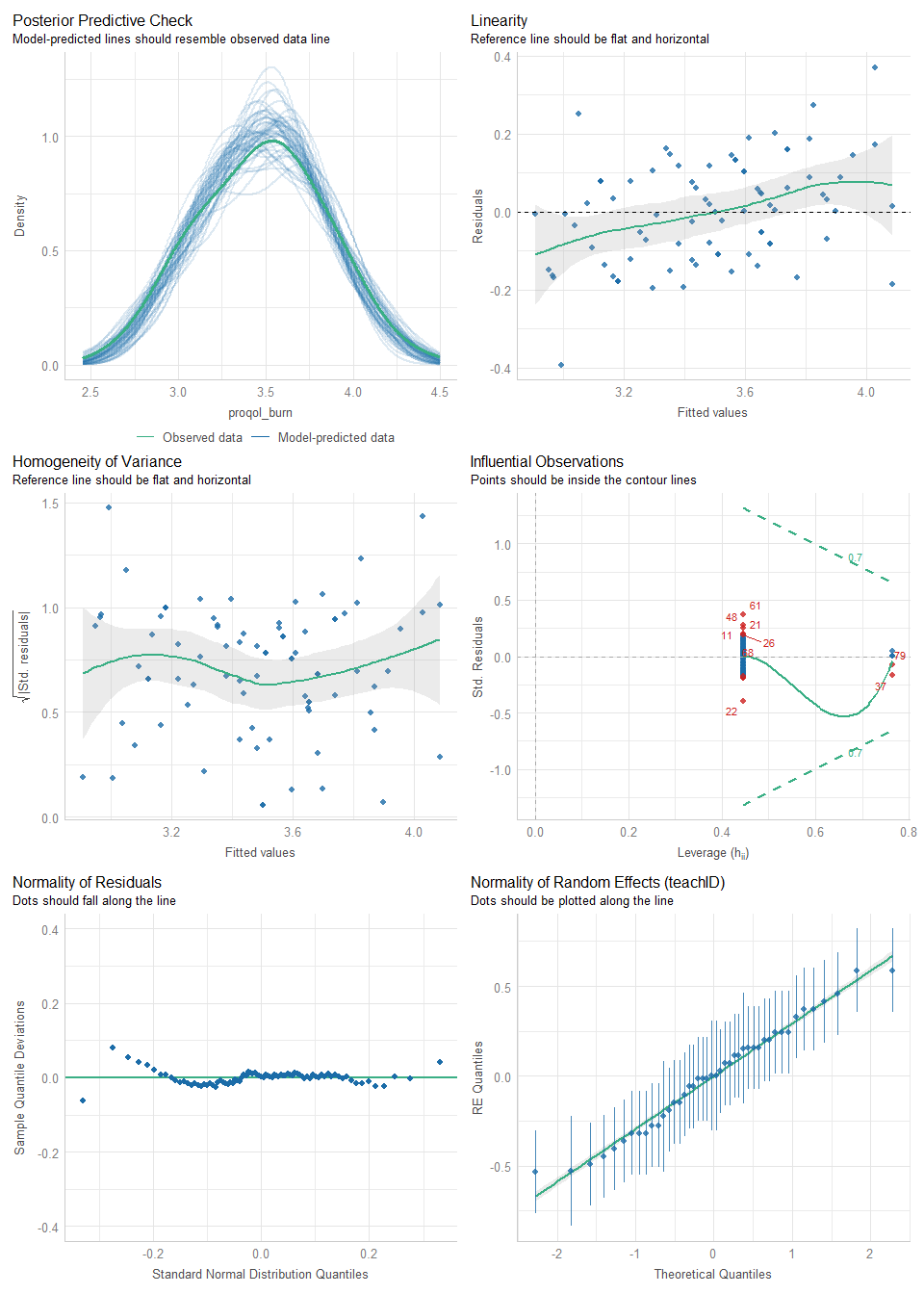


Figure S15: Model diagnostic plots for the ProQOL – Burnout response for the final model for the teachers’ data.

#### Secondary Traumatic Stress

Linear mixed model fit by REML. t-tests use Satterthwaite's method [
lmerModLmerTest]
Formula: proqol_2trauStress ~ time + (1 | teachID)
 Data: proqol_2trauStress_teachData_long_mean

REML criterion at convergence: 108.5

Scaled residuals:
 Min 1Q Median 3Q Max
-1.44395 -0.59946 -0.04295 0.48051 1.63217

Random effects:
 Groups Name Variance Std.Dev.
 teachID (Intercept) 0.17671 0.4204
 Residual 0.09043 0.3007
Number of obs: 83, groups: teachID, 44

Fixed effects:
 Estimate Std. Error df t value Pr(>|t|)
(Intercept) 2.48409 0.07792 57.76661 31.880 < 2e-16 ***
time -0.28941 0.06744 39.29396 -4.291 0.000112 ***
---
Signif. codes: 0 '***' 0.001 '**' 0.01 '*' 0.05 '.' 0.1 ' ' 1

Correlation of Fixed Effects:
 (Intr)
time -0.391

Analysis of Deviance Table (Type II Wald chisquare tests)

Response: proqol_2trauStress
 Chisq Df Pr(>Chisq)
time 18.415 1 1.777e-05 ***
---
Signif. codes: 0 '***' 0.001 '**' 0.01 '*' 0.05 '.' 0.1 ' ' 1


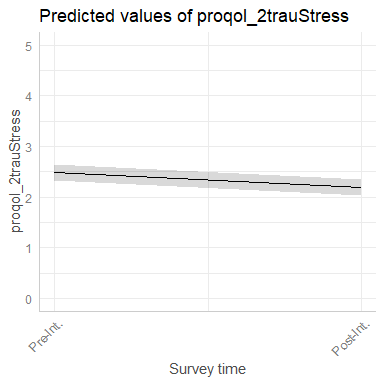


Figure S16: Predicted mean ProQOL Secondary Traumatic Stress score based on the final model that includes time and accounts for the repeated measurements for teachers.

# Predicted values of proqol_2trauStress

time | Predicted | 95% CI
-----------------------------
 0 | 2.48 | 2.33, 2.64
 1 | 2.19 | 2.04, 2.35


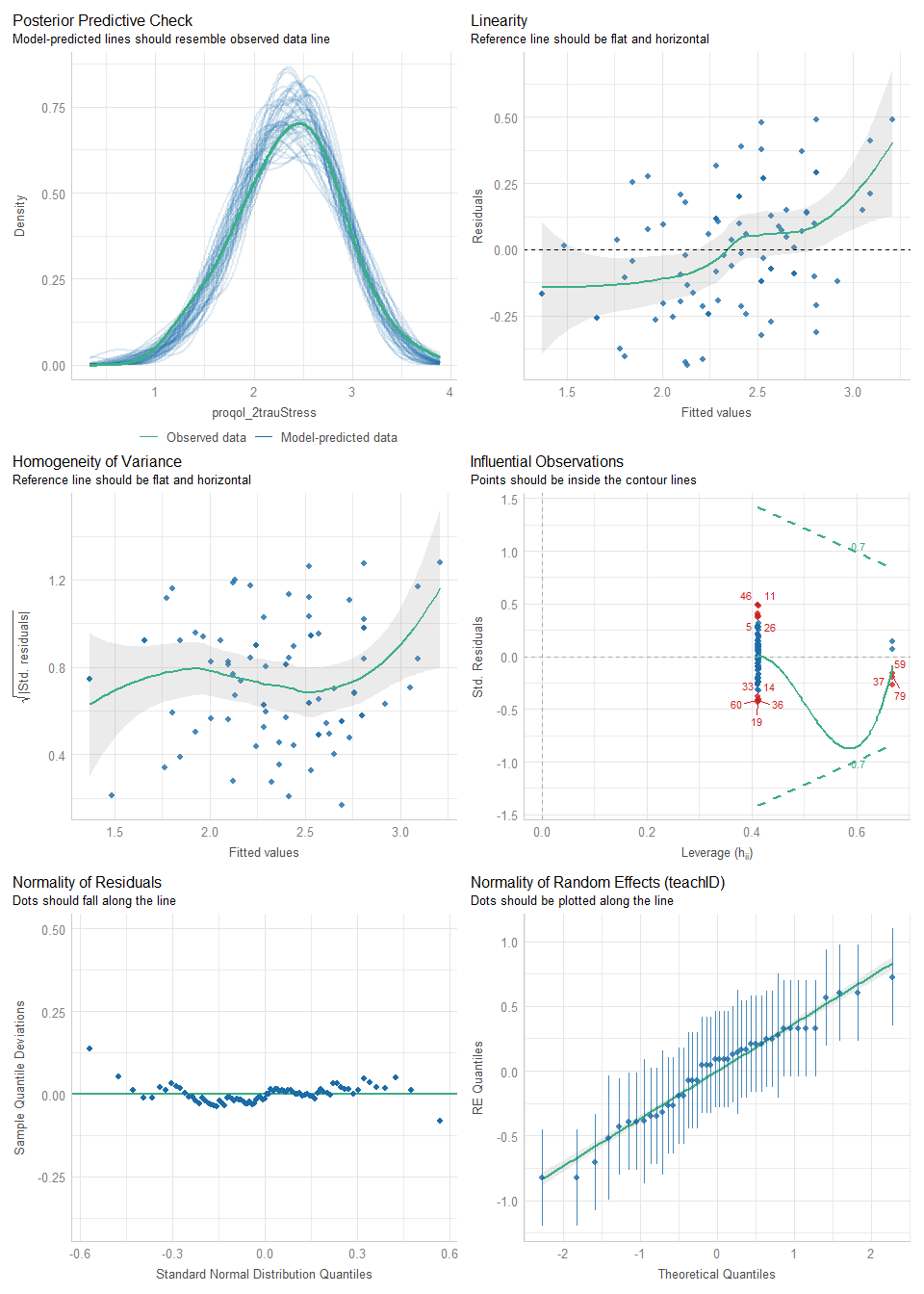


Figure S17: Model diagnostic plots for the ProQOL – Secondary Traumatic Stress response for the final model for the teachers’ data.

### Educator Sense of Self-Efficacy Short Form (TSSE-SF)

#### Student Engagement Final Model

Linear mixed model fit by REML. t-tests use Satterthwaite's method [
lmerModLmerTest]
Formula: teachses_stuEng ~ ace1_1 + time + (1 | teachID)
 Data: teachses_stuEng_teachData_long_mean

REML criterion at convergence: 245.7

Scaled residuals:
 Min 1Q Median 3Q Max
-2.50119 -0.43923 -0.08455 0.45072 2.03422

Random effects:
 Groups Name Variance Std.Dev.
 teachID (Intercept) 0.6481 0.8050
 Residual 0.6004 0.7749
Number of obs: 83, groups: teachID, 44

Fixed effects:
 Estimate Std. Error df t value Pr(>|t|)
(Intercept) 6.01523 0.29127 47.65569 20.652 <2e-16 ***
ace1_1 0.16424 0.07522 41.07498 2.184 0.0348 *
time -0.21770 0.17306 38.73300 -1.258 0.2159
---
Signif. codes: 0 '***' 0.001 '**' 0.01 '*' 0.05 '.' 0.1 ' ' 1

Correlation of Fixed Effects:
 (Intr) ace1_1
ace1_1 -0.816
time -0.267 -0.004

Analysis of Deviance Table (Type II Wald chisquare tests)

Response: teachses_stuEng
 Chisq Df Pr(>Chisq)
ace1_1 4.7678 1 0.0290 *
time 1.5825 1 0.2084
---
Signif. codes: 0 '***' 0.001 '**' 0.01 '*' 0.05 '.' 0.1 ' ' 1


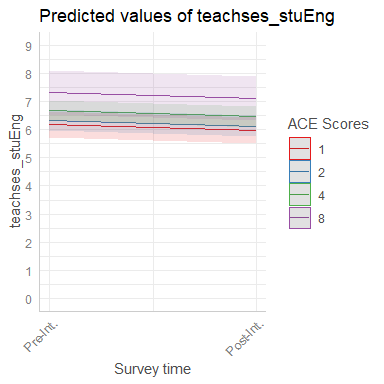


Figure S18: Predicted mean Teachers’ Sense of Efficacy - Efficacy in Student Engagement score based on the final model that includes ACES score, time, number of sessions, and accounts for the repeated measurements for teachers. The predicted line for Teachers’ Sense of Efficacy - Efficacy in Student Engagement by time for an ACES score of 8 for males is based on a prediction assuming a linear relationship between ACES score and Teachers’ Sense of Efficacy - Efficacy in Student Engagement, although the maximum score for males that was observed was 4.

# Predicted values of teachses_stuEng

ace1_1: 1

time | Predicted | 95% CI
-----------------------------
 0 | 6.18 | 5.72, 6.64
 1 | 5.96 | 5.49, 6.43

ace1_1: 2

time | Predicted | 95% CI
-----------------------------
 0 | 6.34 | 5.97, 6.72
 1 | 6.13 | 5.74, 6.51

ace1_1: 4

time | Predicted | 95% CI
-----------------------------
 0 | 6.67 | 6.32, 7.02
 1 | 6.45 | 6.09, 6.82

ace1_1: 8

time | Predicted | 95% CI
-----------------------------
 0 | 7.33 | 6.54, 8.12
 1 | 7.11 | 6.32, 7.90


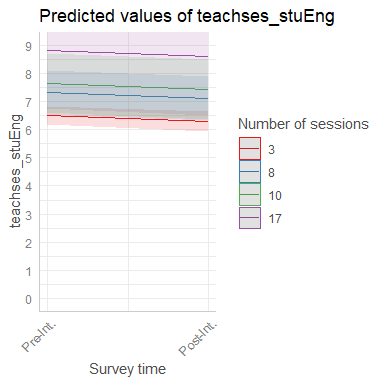


Figure S19: Predicted mean Teachers’ Sense of Efficacy - Efficacy in Student Engagement score based on the final model that includes ACES score, time, number of sessions, and accounts for the repeated measurements for teachers.

# Predicted values of teachses_stuEng

ace1_1: 3

time | Predicted | 95% CI
------------------------------
 0 | 6.51 | 6.18, 6.84
 1 | 6.29 | 5.94, 6.64

ace1_1: 8

time | Predicted | 95% CI
------------------------------
 0 | 7.33 | 6.54, 8.12
 1 | 7.11 | 6.32, 7.90

ace1_1: 10

time | Predicted | 95% CI
------------------------------
 0 | 7.66 | 6.60, 8.72
 1 | 7.44 | 6.38, 8.50

ace1_1: 17

time | Predicted | 95% CI
------------------------------
 0 | 8.81 | 6.74, 10.87
 1 | 8.59 | 6.52, 10.66


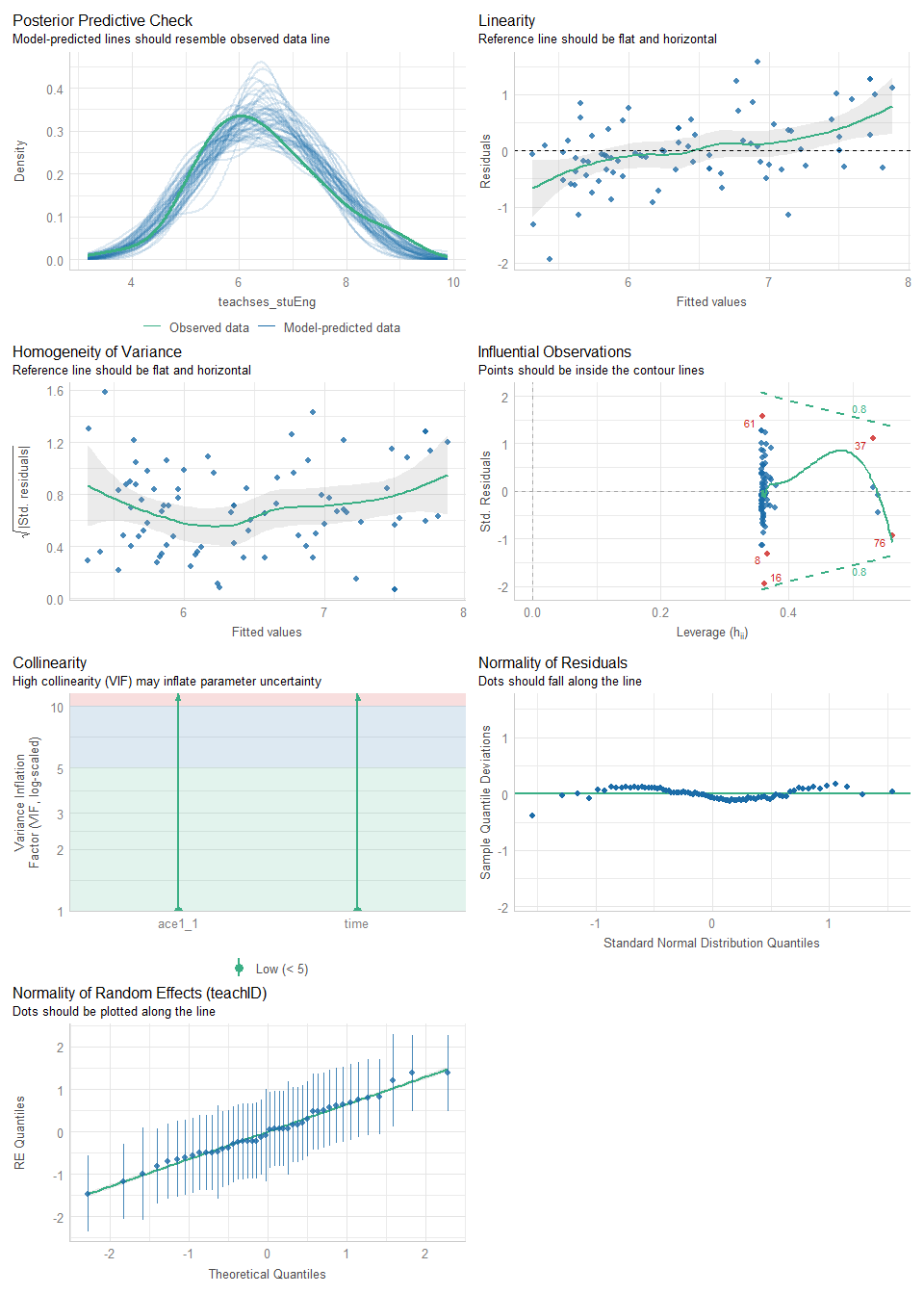


Figure S20: Model diagnostic plots for the TSSE-SF - Efficacy in Student Engagement response for the final model for the teachers’ data.

#### Instructional Strategies

Linear mixed model fit by REML. t-tests use Satterthwaite's method [
lmerModLmerTest]
Formula: teachses_instStrat ~ time + (1 | teachID)
 Data: teachses_instStrat_teachData_long_mean

REML criterion at convergence: 226.2

Scaled residuals:
 Min 1Q Median 3Q Max
-2.59657 -0.54272 0.06039 0.50882 1.73961

Random effects:
 Groups Name Variance Std.Dev.
 teachID (Intercept) 0.7172 0.8468
 Residual 0.4008 0.6331
Number of obs: 83, groups: teachID, 44

Fixed effects:
 Estimate Std. Error df t value Pr(>|t|)
(Intercept) 7.119318 0.159402 59.508252 44.663 <2e-16 ***
time -0.004678 0.141906 40.340319 -0.033 0.974
---
Signif. codes: 0 '***' 0.001 '**' 0.01 '*' 0.05 '.' 0.1 ' ' 1

Correlation of Fixed Effects:
 (Intr)
time -0.403

Analysis of Deviance Table (Type II Wald chisquare tests)

Response: teachses_instStrat
 Chisq Df Pr(>Chisq)
time 0.0011 1 0.9737


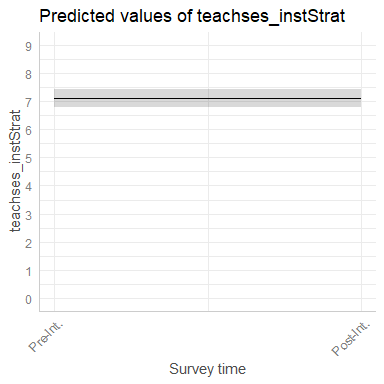


Figure S21: Predicted mean Teachers’ Sense of Efficacy - Efficacy in Instructional Strategies score based on the final model that includes time and accounts for the repeated measurements for teachers.

# Predicted values of teachses_instStrat

time | Predicted | 95% CI
-----------------------------
 0 | 7.12 | 6.81, 7.43
 1 | 7.11 | 6.79, 7.44


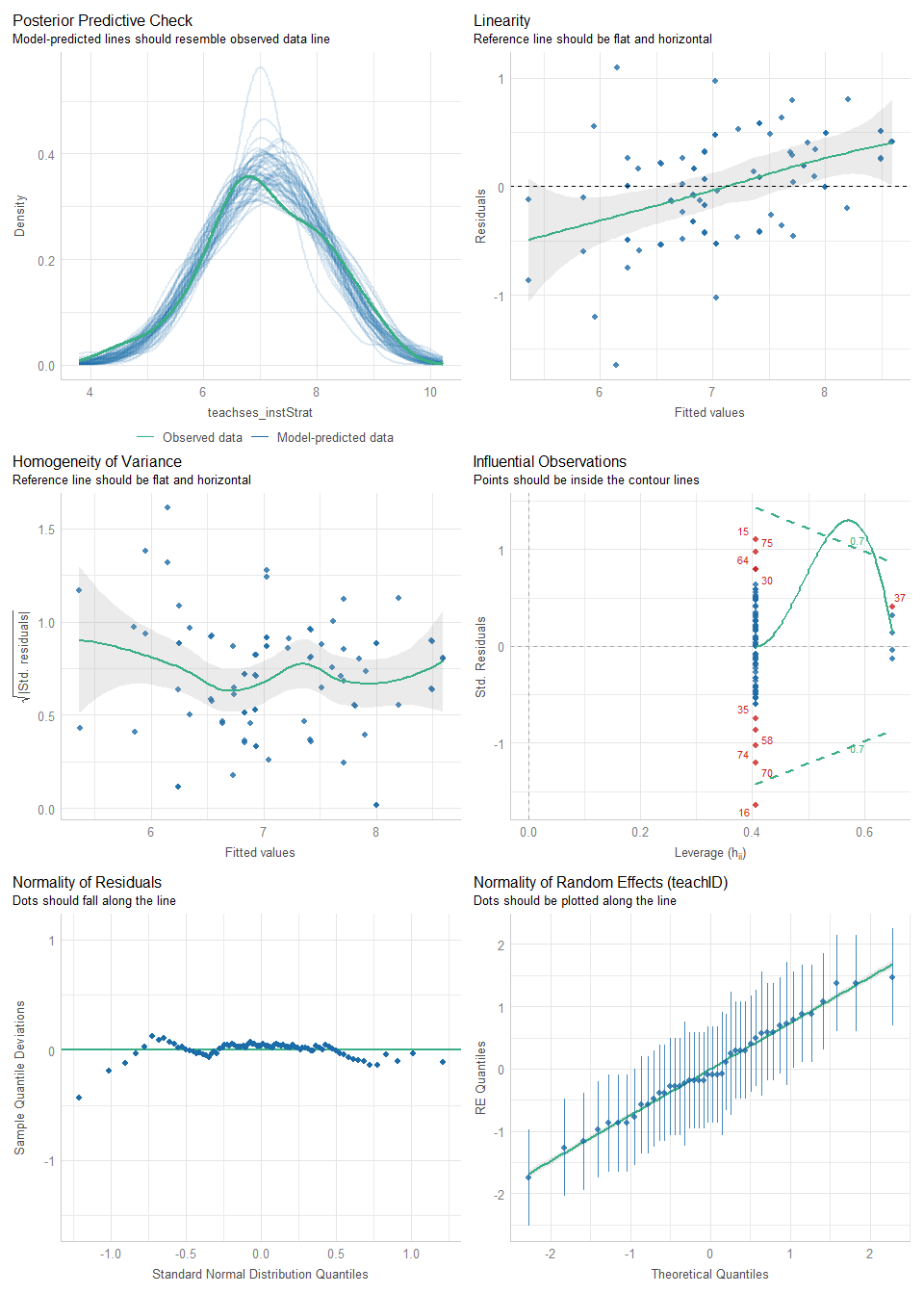


Figure S22: Model diagnostic plots for the TSSE-SF - Efficacy in Instructional Strategies response for the final model for the teachers’ data.

#### Classroom Management

Linear mixed model fit by REML. t-tests use Satterthwaite's method [
lmerModLmerTest]
Formula: teachses_clMan ~ ace1_1 + time + (1 | teachID)
 Data: teachses_clMan_teachData_long_mean

REML criterion at convergence: 227.2

Scaled residuals:
 Min 1Q Median 3Q Max
-2.08702 -0.40755 0.05507 0.35786 1.84732

Random effects:
 Groups Name Variance Std.Dev.
 teachID (Intercept) 0.8516 0.9228
 Residual 0.3445 0.5869
Number of obs: 83, groups: teachID, 44

Fixed effects:
 Estimate Std. Error df t value Pr(>|t|)
(Intercept) 6.63103 0.29537 46.50072 22.450 <2e-16 ***
ace1_1 0.16176 0.07758 43.05102 2.085 0.043 *
time -0.13965 0.13182 39.97860 -1.059 0.296
---
Signif. codes: 0 '***' 0.001 '**' 0.01 '*' 0.05 '.' 0.1 ' ' 1

Correlation of Fixed Effects:
 (Intr) ace1_1
ace1_1 -0.830
time -0.198 -0.003

Analysis of Deviance Table (Type II Wald chisquare tests)

Response: teachses_clMan
 Chisq Df Pr(>Chisq)
ace1_1 4.3479 1 0.03705 *
time 1.1224 1 0.28939
---
Signif. codes: 0 '***' 0.001 '**' 0.01 '*' 0.05 '.' 0.1 ' ' 1


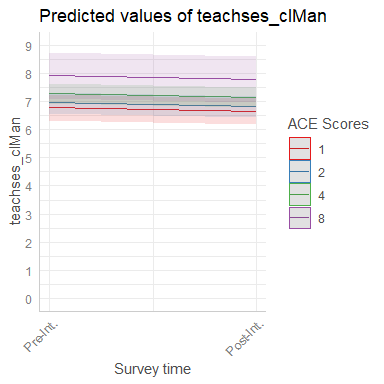


Figure S23: Predicted mean Teachers’ Sense of Efficacy - Efficacy in Classroom Management score based on the final model that includes ACES score, time, and accounts for the repeated measurements for teachers. The predicted line for Teachers’ Sense of Efficacy - Efficacy in Classroom Management by time for an ACES score of 8 for males is based on a prediction assuming a linear relationship between ACES score and Teachers’ Sense of Efficacy - Efficacy in Classroom Management, although the maximum score for males that was observed was 4.

# Predicted values of teachses_clMan

ace1_1: 1

time | Predicted | 95% CI
-----------------------------
 0 | 6.79 | 6.33, 7.25
 1 | 6.65 | 6.18, 7.12

ace1_1: 2

time | Predicted | 95% CI
-----------------------------
 0 | 6.95 | 6.59, 7.32
 1 | 6.81 | 6.44, 7.19

ace1_1: 4

time | Predicted | 95% CI
-----------------------------
 0 | 7.28 | 6.93, 7.63
 1 | 7.14 | 6.78, 7.49

ace1_1: 8

time | Predicted | 95% CI
-----------------------------
 0 | 7.93 | 7.12, 8.73
 1 | 7.79 | 6.98, 8.59


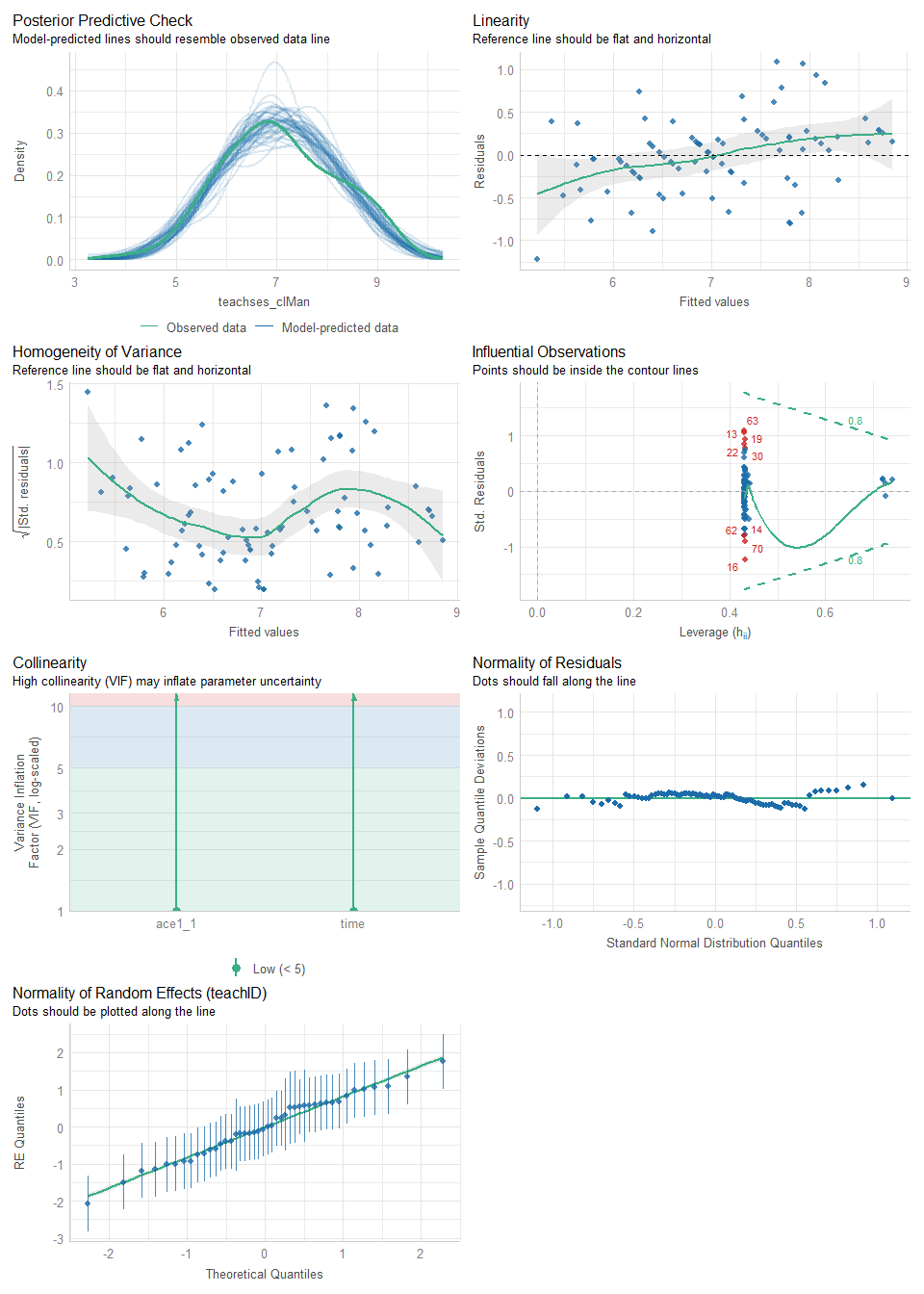


Figure S24: Model diagnostic plots for the TSSE-SF - Efficacy in Classroom Management response for the final model for the teachers’ data.

## Student models

### Generalized Anxiety Disorder – 7 (GAD-7-Child) Final Model

Linear mixed model fit by REML. t-tests use Satterthwaite's method [
lmerModLmerTest]
Formula: gad7 ~ ace1_1 + time + gen_cnf + ace1_1:time + (1 | studentID)
 Data: gad7_studentData_long_mean

REML criterion at convergence: 147.5

Scaled residuals:
 Min 1Q Median 3Q Max
-2.20294 -0.54488 -0.08982 0.53277 2.41318

Random effects:
 Groups Name Variance Std.Dev.
 studentID (Intercept) 0.1212 0.3481
 Residual 0.2450 0.4950
Number of obs: 76, groups: studentID, 40

Fixed effects:
 Estimate Std. Error df t value Pr(>|t|)
(Intercept) 0.33460 0.18844 61.50880 1.776 0.08073 .
ace1_1 0.15231 0.03559 61.89797 4.280 6.61e-05 ***
time 0.15852 0.21767 34.11895 0.728 0.47142
gen_cnfFemale 0.47478 0.16475 34.83241 2.882 0.00673 **
ace1_1:time -0.08649 0.04084 33.47343 -2.118 0.04170 *
---
Signif. codes: 0 '***' 0.001 '**' 0.01 '*' 0.05 '.' 0.1 ' ' 1

Correlation of Fixed Effects:
 (Intr) ace1_1 time gn_cnF
ace1_1 -0.749
time -0.544 0.465
gen_cnfFeml -0.251 -0.221 0.004
ace1_1:time 0.469 -0.554 -0.850 -0.002


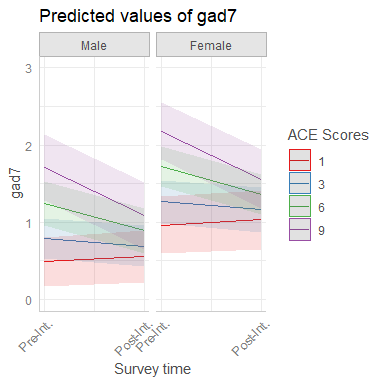


Figure S25: Predicted mean GAD-7 score based on the final model that includes ACES score, timing of the survey, gender, and accounts for the repeated measurements for students.

# Predicted values of gad7

ace1_1: 1
gen_cnf: Male

time | Predicted | 95% CI
-----------------------------
 0 | 0.49 | 0.17, 0.81
 1 | 0.56 | 0.23, 0.89

ace1_1: 1
gen_cnf: Female

time | Predicted | 95% CI
-----------------------------
 0 | 0.96 | 0.59, 1.33
 1 | 1.03 | 0.65, 1.42

ace1_1: 3
gen_cnf: Male

time | Predicted | 95% CI
-----------------------------
 0 | 0.79 | 0.54, 1.05
 1 | 0.69 | 0.43, 0.95

ace1_1: 3
gen_cnf: Female

time | Predicted | 95% CI
-----------------------------
 0 | 1.27 | 0.98, 1.55
 1 | 1.17 | 0.87, 1.46

ace1_1: 6
gen_cnf: Male

time | Predicted | 95% CI
-----------------------------
 0 | 1.25 | 0.97, 1.53
 1 | 0.89 | 0.60, 1.18

ace1_1: 6
gen_cnf: Female

time | Predicted | 95% CI
-----------------------------
 0 | 1.72 | 1.47, 1.98
 1 | 1.36 | 1.10, 1.62

ace1_1: 9
gen_cnf: Male

time | Predicted | 95% CI
-----------------------------
 0 | 1.71 | 1.28, 2.13
 1 | 1.09 | 0.65, 1.52

ace1_1: 9
gen_cnf: Female

time | Predicted | 95% CI
-----------------------------
 0 | 2.18 | 1.81, 2.55
 1 | 1.56 | 1.18, 1.94


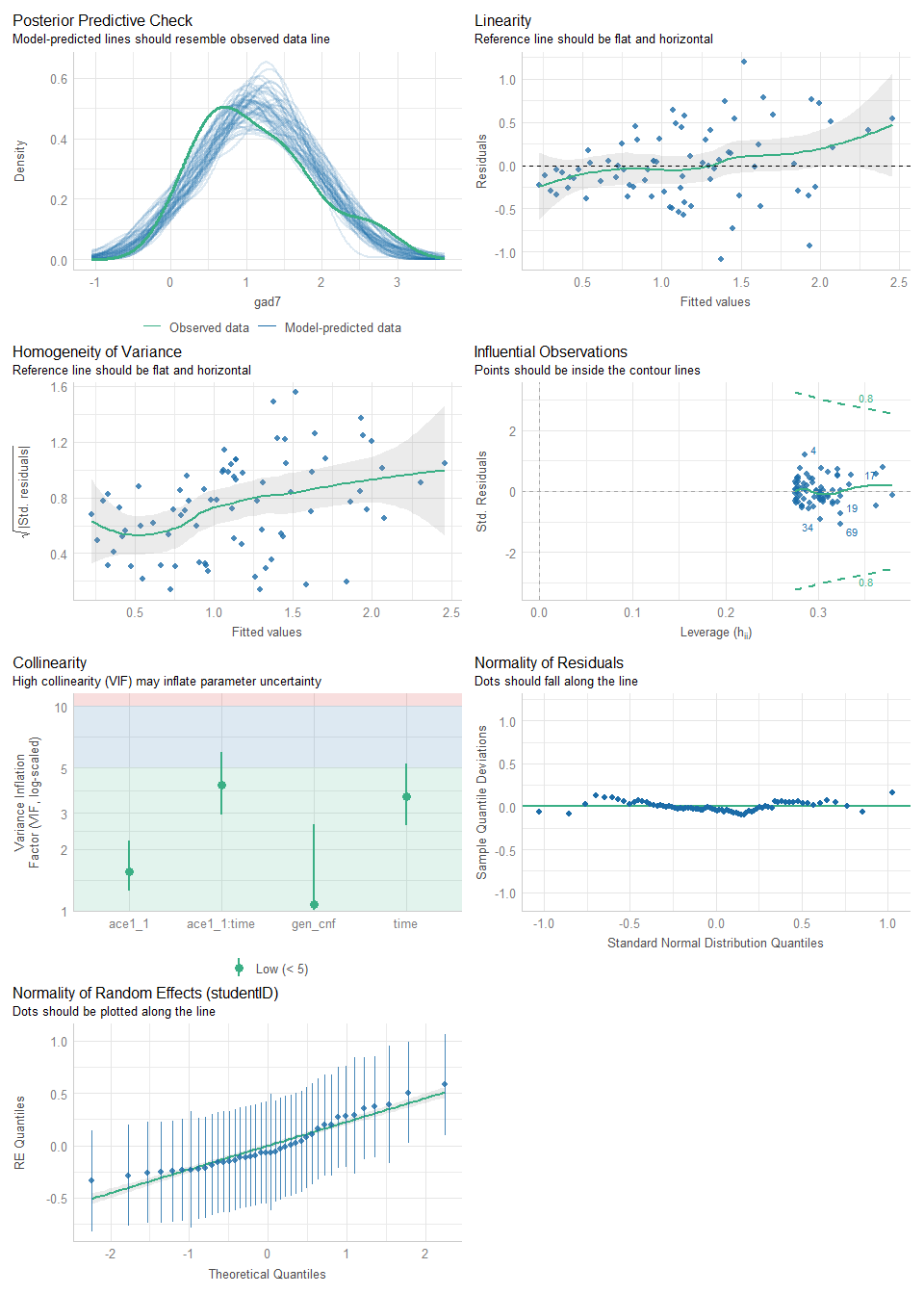


Figure S26: Model diagnostic plot for the GAD-7 response for the students’ data.

### Patient Health Questionnaire – A (PHQ-A) Final Model

Linear mixed model fit by REML. t-tests use Satterthwaite's method [
lmerModLmerTest]
Formula: phq9 ~ ace1_1 + time + gen_cnf + district + ace1_1:gen_cnf +
 ace1_1:district + gen_cnf:district + ace1_1:gen_cnf:district +
 (1 | studentID)
 Data: phq9_studentData_long_mean

REML criterion at convergence: 134.8

Scaled residuals:
 Min 1Q Median 3Q Max
-2.31471 -0.49846 -0.03218 0.45158 2.89505

Random effects:
 Groups Name Variance Std.Dev.
 studentID (Intercept) 0.08727 0.2954
 Residual 0.19756 0.4445
Number of obs: 76, groups: studentID, 40

Fixed effects:
 Estimate Std. Error df t value Pr(>|t|)
(Intercept) -0.07581 0.32065 36.99253 -0.236 0.814398
ace1_1 0.25350 0.06494 34.36808 3.904 0.000421
time -0.30613 0.10316 37.43095 -2.968 0.005206
gen_cnfFemale 1.36986 0.43954 34.41484 3.117 0.003681
districtL 0.59602 0.39358 34.05599 1.514 0.139164
ace1_1:gen_cnfFemale -0.20716 0.08178 33.13698 -2.533 0.016211
ace1_1:districtL -0.22905 0.08806 33.64340 -2.601 0.013712
gen_cnfFemale:districtL -1.24058 0.56166 32.83741 -2.209 0.034281
ace1_1:gen_cnfFemale:districtL 0.25072 0.11026 32.57695 2.274 0.029702

(Intercept)
ace1_1 ***
time **
gen_cnfFemale **
districtL
ace1_1:gen_cnfFemale *
ace1_1:districtL *
gen_cnfFemale:districtL *
ace1_1:gen_cnfFemale:districtL *
---
Signif. codes: 0 '***' 0.001 '**' 0.01 '*' 0.05 '.' 0.1 ' ' 1

Correlation of Fixed Effects:
 (Intr) ace1_1 time gn_cnF dstrcL ac1_1:_F a1_1:L gn_F:L
ace1_1 -0.889
time -0.114 -0.040
gen_cnfFeml -0.720 0.652 -0.003
districtL -0.799 0.730 -0.044 0.587
ac1_1:gn_cF 0.708 -0.793 0.016 -0.893 -0.579
ac1_1:dstrL 0.653 -0.738 0.051 -0.481 -0.854 0.585
gn_cnfFml:L 0.563 -0.510 0.006 -0.783 -0.700 0.699 0.597
ac1_1:g_F:L -0.523 0.589 -0.029 0.663 0.682 -0.742 -0.798 -0.851


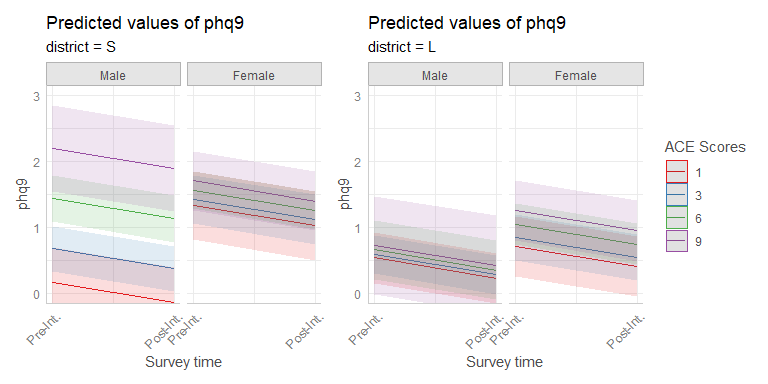


Figure S27: Predicted mean PHQ-9 score based on the final model that includes a three-way interaction between ACES score, gender, and district, as well as timing of the survey, and accounts for the repeated measurements for students.

[1] "### district == 'S'"

# Predicted values of phq9

ace1_1: 1
gen_cnf: Male

time | Predicted | 95% CI
------------------------------
 0 | 0.18 | -0.34, 0.70
 1 | -0.13 | -0.66, 0.40

ace1_1: 1
gen_cnf: Female

time | Predicted | 95% CI
-----------------------------
 0 | 1.34 | 0.83, 1.85
 1 | 1.03 | 0.51, 1.56

ace1_1: 3
gen_cnf: Male

time | Predicted | 95% CI
-----------------------------
 0 | 0.68 | 0.35, 1.02
 1 | 0.38 | 0.03, 0.72

ace1_1: 3
gen_cnf: Female

time | Predicted | 95% CI
-----------------------------
 0 | 1.43 | 1.07, 1.80
 1 | 1.13 | 0.75, 1.50

ace1_1: 6
gen_cnf: Male

time | Predicted | 95% CI
-----------------------------
 0 | 1.45 | 1.09, 1.80
 1 | 1.14 | 0.79, 1.49

ace1_1: 6
gen_cnf: Female

time | Predicted | 95% CI
-----------------------------
 0 | 1.57 | 1.29, 1.86
 1 | 1.27 | 0.97, 1.56

ace1_1: 9
gen_cnf: Male

time | Predicted | 95% CI
-----------------------------
 0 | 2.21 | 1.55, 2.86
 1 | 1.90 | 1.25, 2.55

ace1_1: 9
gen_cnf: Female

time | Predicted | 95% CI
-----------------------------
 0 | 1.71 | 1.27, 2.16
 1 | 1.40 | 0.96, 1.85

[1] "### district == 'L'"

# Predicted values of phq9

ace1_1: 1
gen_cnf: Male

time | Predicted | 95% CI
------------------------------
 0 | 0.54 | 0.17, 0.92
 1 | 0.24 | -0.14, 0.62

ace1_1: 1
gen_cnf: Female

time | Predicted | 95% CI
------------------------------
 0 | 0.72 | 0.26, 1.17
 1 | 0.41 | -0.05, 0.87

ace1_1: 3
gen_cnf: Male

time | Predicted | 95% CI
------------------------------
 0 | 0.59 | 0.31, 0.88
 1 | 0.29 | -0.01, 0.58

ace1_1: 3
gen_cnf: Female

time | Predicted | 95% CI
-----------------------------
 0 | 0.85 | 0.51, 1.20
 1 | 0.55 | 0.20, 0.89

ace1_1: 6
gen_cnf: Male

time | Predicted | 95% CI
------------------------------
 0 | 0.67 | 0.23, 1.11
 1 | 0.36 | -0.09, 0.81

ace1_1: 6
gen_cnf: Female

time | Predicted | 95% CI
-----------------------------
 0 | 1.06 | 0.75, 1.37
 1 | 0.75 | 0.44, 1.06

ace1_1: 9
gen_cnf: Male

time | Predicted | 95% CI
------------------------------
 0 | 0.74 | 0.00, 1.48
 1 | 0.43 | -0.32, 1.18

ace1_1: 9
gen_cnf: Female

time | Predicted | 95% CI
-----------------------------
 0 | 1.26 | 0.80, 1.72
 1 | 0.96 | 0.50, 1.41


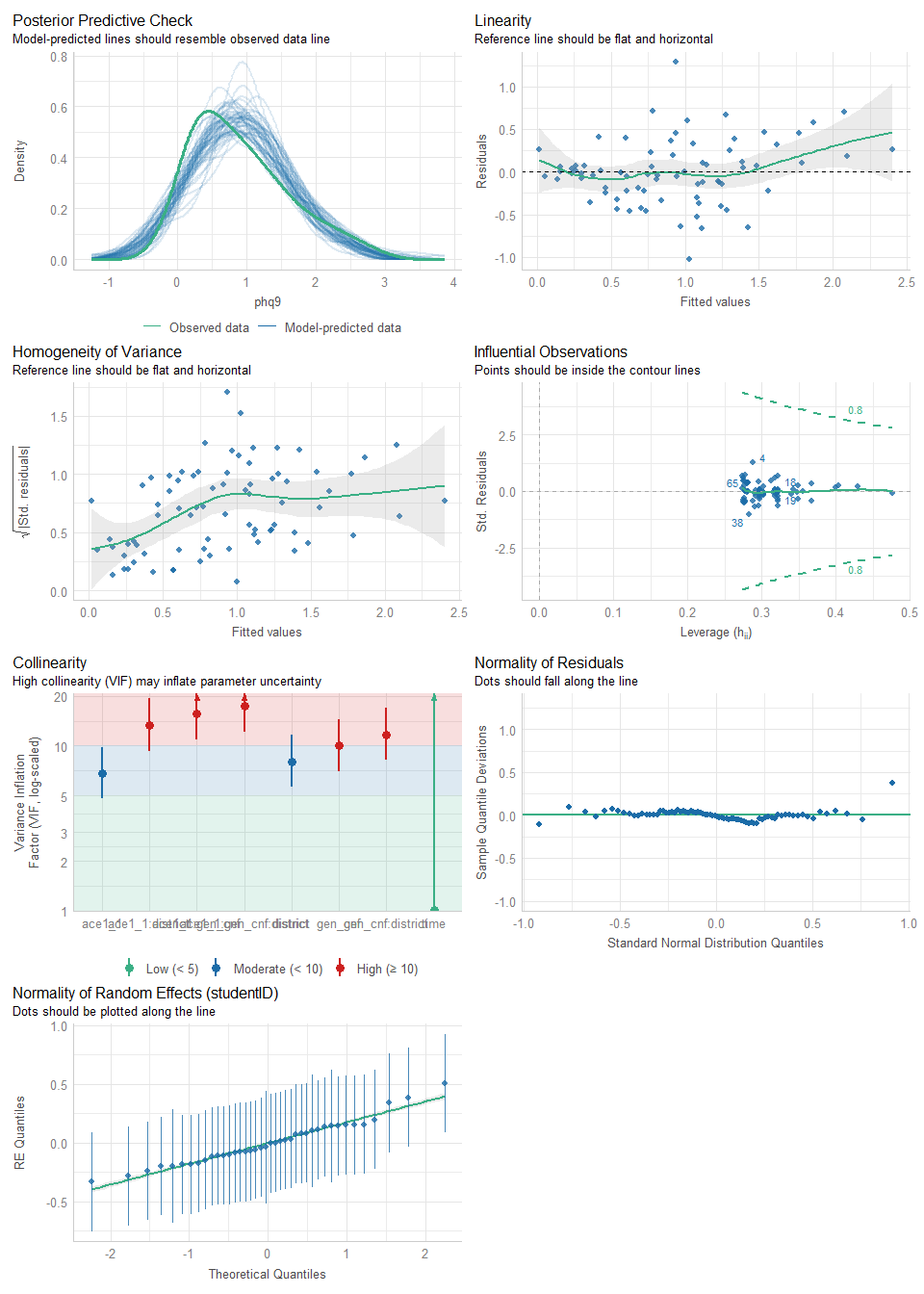


Figure S28: Model diagnostic plot for the PHQ-9 response for the students’ data.

### Patient-Reported Outcomes Measurement Information System – Sleep Disturbance Short Form for Children Ages 11-17 (PROMIS SD-SF-Child) Final Model

Linear mixed model fit by REML. t-tests use Satterthwaite's method [
lmerModLmerTest]
Formula: promsd ~ age + ace1_1 + time + district + ace1_1:district + (1 |
 studentID)
 Data: promsd_studentData_long_mean

REML criterion at convergence: 201.5

Scaled residuals:
 Min 1Q Median 3Q Max
-2.89174 -0.36835 0.02289 0.37453 3.02304

Random effects:
 Groups Name Variance Std.Dev.
 studentID (Intercept) 0.5798 0.7615
 Residual 0.3387 0.5819
Number of obs: 77, groups: studentID, 41

Fixed effects:
 Estimate Std. Error df t value Pr(>|t|)
(Intercept) -3.17952 2.60017 36.41577 -1.223 0.22926
age 0.33017 0.15691 36.19579 2.104 0.04238 *
ace1_1 0.25272 0.07703 36.43633 3.281 0.00229 **
time -0.43292 0.13579 36.47479 -3.188 0.00294 **
districtL 0.79344 0.53989 36.55816 1.470 0.15021
ace1_1:districtL -0.24291 0.10147 35.66663 -2.394 0.02206 *
---
Signif. codes: 0 '***' 0.001 '**' 0.01 '*' 0.05 '.' 0.1 ' ' 1

Correlation of Fixed Effects:
 (Intr) age ace1_1 time dstrcL
age -0.987
ace1_1 -0.309 0.173
time -0.006 -0.011 -0.042
districtL -0.285 0.167 0.699 -0.048
ac1_1:dstrL 0.157 -0.053 -0.746 0.038 -0.850


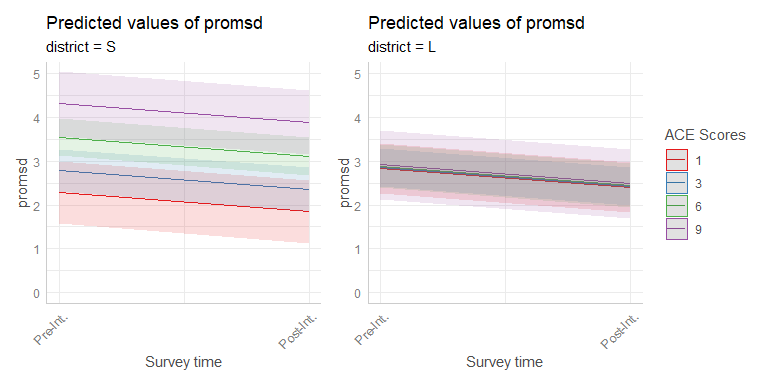


Figure S29: Predicted mean PROMIS score based on the final model that includes a two-way interaction between ACES score and district, as well as timing of the survey, age, and accounts for the repeated measurements for students.

# Predicted values of promsd

ace1_1: 1

time | Predicted | 95% CI
-----------------------------
 0 | 2.28 | 1.58, 2.98
 1 | 1.85 | 1.13, 2.57

ace1_1: 3

time | Predicted | 95% CI
-----------------------------
 0 | 2.79 | 2.31, 3.27
 1 | 2.36 | 1.86, 2.85

ace1_1: 6

time | Predicted | 95% CI
-----------------------------
 0 | 3.55 | 3.12, 3.97
 1 | 3.11 | 2.68, 3.55

ace1_1: 9

time | Predicted | 95% CI
-----------------------------
 0 | 4.30 | 3.57, 5.04
 1 | 3.87 | 3.14, 4.61

Adjusted for:
* age = 15.78

# Predicted values of promsd

ace1_1: 1

time | Predicted | 95% CI
-----------------------------
 0 | 2.83 | 2.26, 3.41
 1 | 2.40 | 1.83, 2.98

ace1_1: 3

time | Predicted | 95% CI
-----------------------------
 0 | 2.85 | 2.42, 3.29
 1 | 2.42 | 1.98, 2.86

ace1_1: 6

time | Predicted | 95% CI
-----------------------------
 0 | 2.88 | 2.39, 3.38
 1 | 2.45 | 1.95, 2.95

ace1_1: 9

time | Predicted | 95% CI
-----------------------------
 0 | 2.91 | 2.13, 3.70
 1 | 2.48 | 1.69, 3.27

Adjusted for:
* age = 15.78


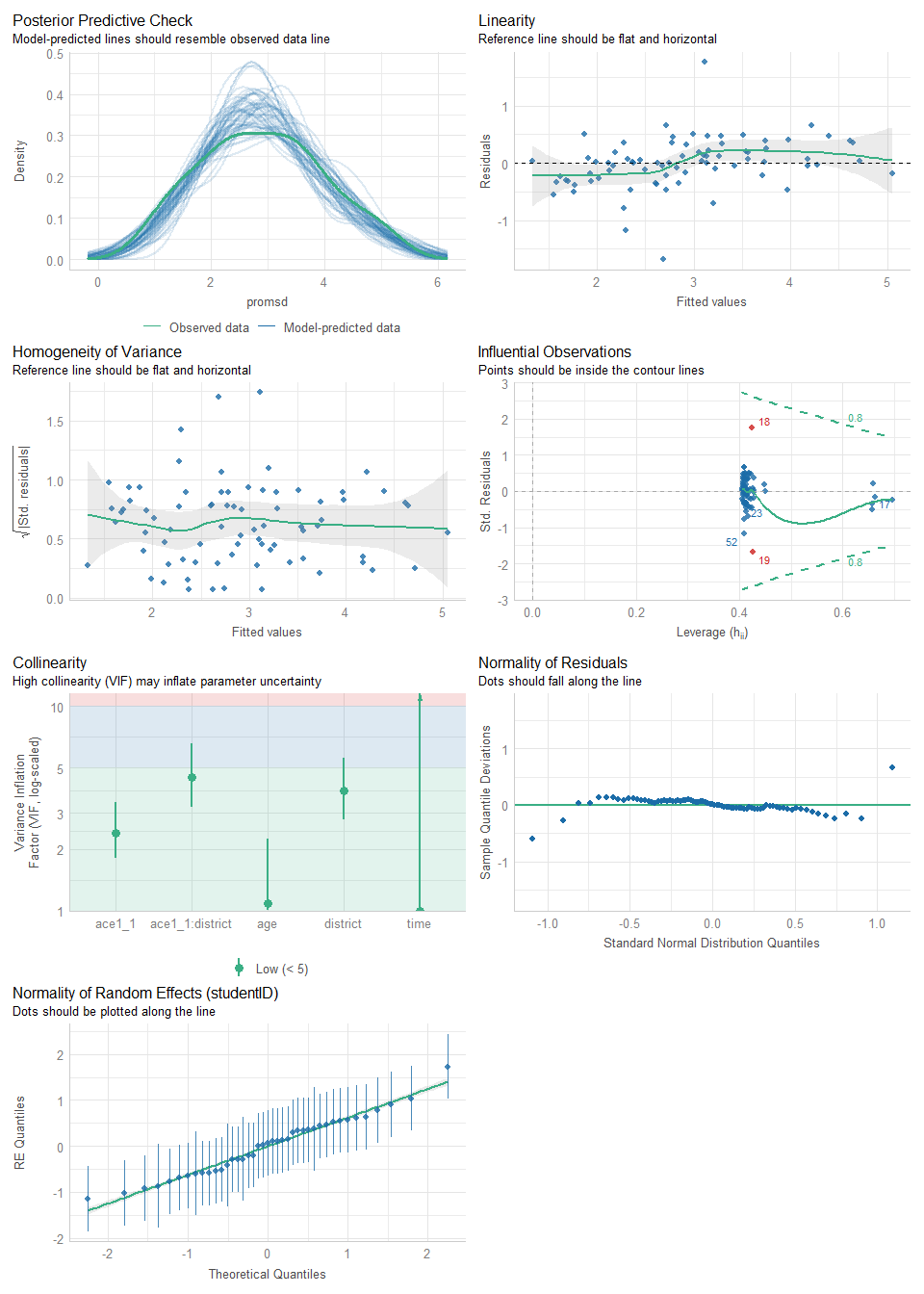


Figure S30: Model diagnostic plot for the PROMIS SD response for the students’ data.

### Connor Davidson Resilience Scale (CD-RISC) Final Model

Linear mixed model fit by REML. t-tests use Satterthwaite's method [
lmerModLmerTest]
Formula: cdrisc ~ ace1_1 + time + gen_cnf + ace1_1:gen_cnf + (1 | studentID)
 Data: cdrisc_studentData_long_mean

REML criterion at convergence: 128.5

Scaled residuals:
 Min 1Q Median 3Q Max
-2.0187 -0.3857 0.0634 0.3600 1.8512

Random effects:
 Groups Name Variance Std.Dev.
 studentID (Intercept) 0.34775 0.5897
 Residual 0.09203 0.3034
Number of obs: 76, groups: studentID, 40

Fixed effects:
 Estimate Std. Error df t value Pr(>|t|)
(Intercept) 3.38613 0.26842 36.76959 12.615 6.32e-15 ***
ace1_1 -0.14453 0.06026 35.69435 -2.398 0.02183 *
time 0.04058 0.07113 35.37262 0.571 0.57191
gen_cnfFemale -1.22593 0.39092 35.55436 -3.136 0.00343 **
ace1_1:gen_cnfFemale 0.18888 0.07687 35.43439 2.457 0.01904 *
---
Signif. codes: 0 '***' 0.001 '**' 0.01 '*' 0.05 '.' 0.1 ' ' 1

Correlation of Fixed Effects:
 (Intr) ace1_1 time gn_cnF
ace1_1 -0.842
time -0.118 -0.002
gen_cnfFeml -0.678 0.578 0.005
ac1_1:gn_cF 0.661 -0.784 -0.004 -0.849


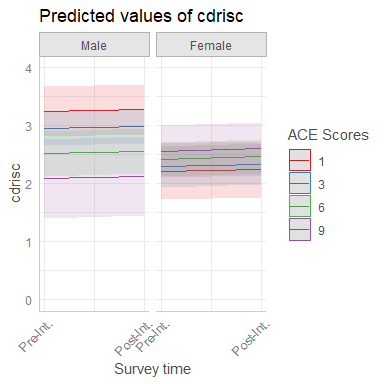


Figure S31: Predicted mean CD-RISC score based on the final model that includes ACES score, timing of the survey, gender, and accounts for the repeated measurements for students.

[1] "### EMMEANS tables for time by ACE Score by Gender"

# Predicted values of cdrisc

ace1_1: 1
gen_cnf: Male

time | Predicted | 95% CI
-----------------------------
 0 | 3.24 | 2.81, 3.67
 1 | 3.28 | 2.85, 3.72

ace1_1: 1
gen_cnf: Female

time | Predicted | 95% CI
-----------------------------
 0 | 2.20 | 1.72, 2.69
 1 | 2.25 | 1.76, 2.73

ace1_1: 3
gen_cnf: Male

time | Predicted | 95% CI
-----------------------------
 0 | 2.95 | 2.66, 3.25
 1 | 2.99 | 2.69, 3.29

ace1_1: 3
gen_cnf: Female

time | Predicted | 95% CI
-----------------------------
 0 | 2.29 | 1.94, 2.64
 1 | 2.33 | 1.98, 2.69

ace1_1: 6
gen_cnf: Male

time | Predicted | 95% CI
-----------------------------
 0 | 2.52 | 2.13, 2.91
 1 | 2.56 | 2.17, 2.95

ace1_1: 6
gen_cnf: Female

time | Predicted | 95% CI
-----------------------------
 0 | 2.43 | 2.13, 2.72
 1 | 2.47 | 2.17, 2.76

ace1_1: 9
gen_cnf: Male

time | Predicted | 95% CI
-----------------------------
 0 | 2.09 | 1.40, 2.77
 1 | 2.13 | 1.44, 2.81

ace1_1: 9
gen_cnf: Female

time | Predicted | 95% CI
-----------------------------
 0 | 2.56 | 2.11, 3.01
 1 | 2.60 | 2.14, 3.06

[1] "### EMMEANS tables for ACE Score by Gender"

# Predicted values of cdrisc

gen_cnf: Male

ace1_1 | Predicted | 95% CI
-------------------------------
 1 | 3.26 | 2.83, 3.69
 2 | 3.12 | 2.77, 3.46
 4 | 2.83 | 2.55, 3.11
 5 | 2.68 | 2.37, 3.00
 6 | 2.54 | 2.15, 2.92
 9 | 2.10 | 1.43, 2.78

gen_cnf: Female

ace1_1 | Predicted | 95% CI
-------------------------------
 1 | 2.22 | 1.74, 2.71
 2 | 2.27 | 1.86, 2.68
 4 | 2.36 | 2.06, 2.66
 5 | 2.40 | 2.12, 2.68
 6 | 2.45 | 2.16, 2.73
 9 | 2.58 | 2.13, 3.03

Adjusted for:
* time = 0.47


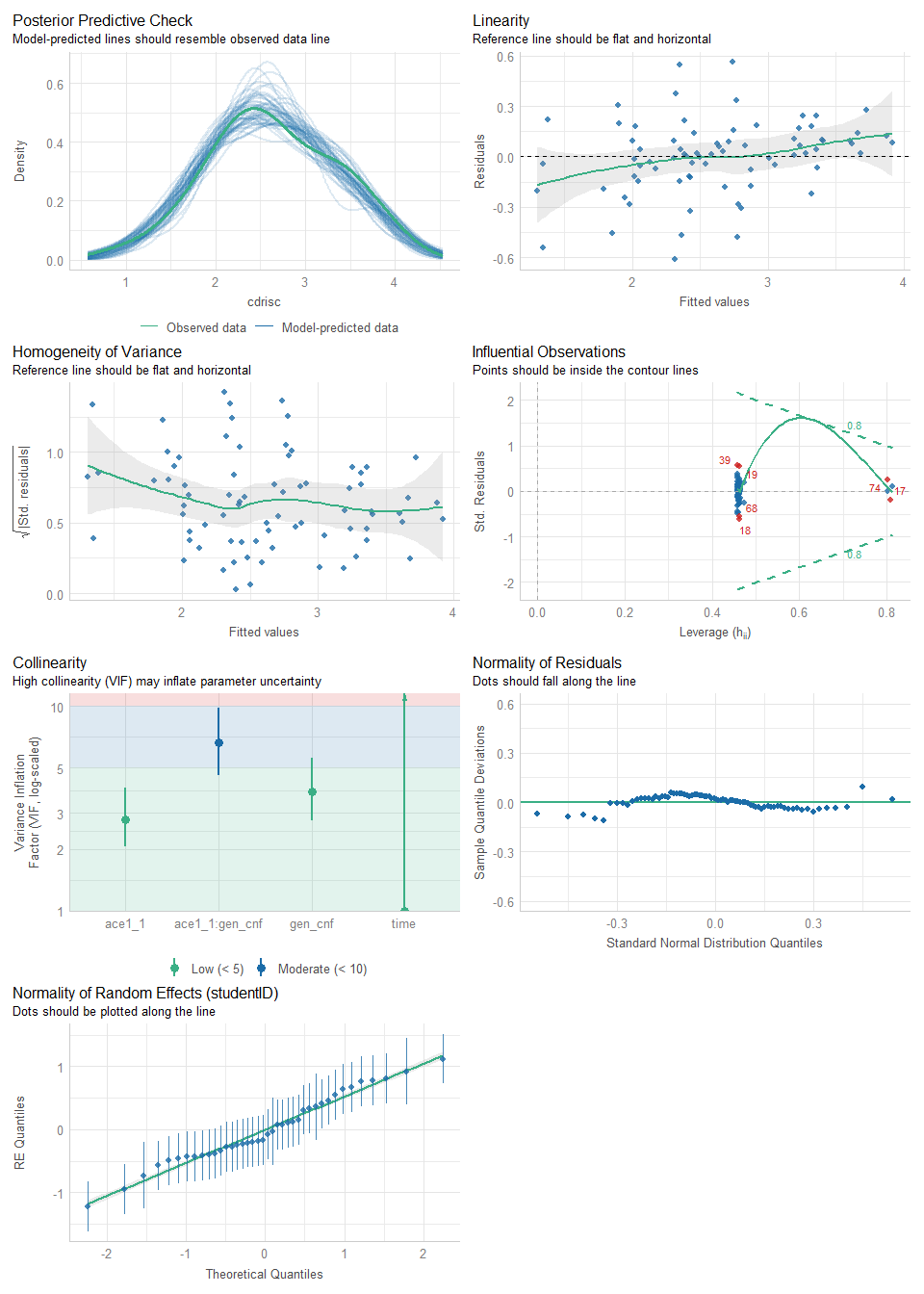


Figure S32: Model diagnostic plot for the CD-RISC response for the students’ data.
